# Antiseizure Medication-Induced Alopecia: A Literature Review

**DOI:** 10.3390/medicines10060035

**Published:** 2023-06-09

**Authors:** Jamir Pitton Rissardo, Ana Leticia Fornari Caprara, Maritsa Casares, Holly J. Skinner, Umair Hamid

**Affiliations:** 1 Medicine Department, Federal University of Santa Maria, Santa Maria 97105-900, Brazil; jamirrissardo@gmail.com (J.P.R.); ana.leticia.fornari@gmail.com (A.L.F.C.); 2 AdventHealth Orlando Neuroscience Institute, 615 E Princeton Street, Suite 540, Orlando, FL 32803, USA; maritsa.casares@adventhealth.com; 3 AdventHealth Epilepsy at Orlando, 615 E Princeton Street, Suite 540, Orlando, FL 32803, USA; holly.skinner.do@adventhealth.com; 4 Department of Neurology, College of Medicine, University of Illinois, Peoria, IL 61605, USA

**Keywords:** alopecia, hair loss, cosmetic side effects, antiseizure medication, anticonvulsant

## Abstract

**Background:** Adverse effects of antiseizure medications (ASMs) remain one of the major causes of non-adherence. Cosmetic side effects (CSEs) are among the most commonly reported side effects of ASMs. In this context, alopecia is one of the CSEs that has a high intolerance rate leading to poor therapeutical compliance. **Methods:** We performed a literature review concerning alopecia as a secondary effect of ASMs. **Results:** There are 1656 individuals reported with ASM-induced alopecia. Valproate (983), lamotrigine (355), and carbamazepine (225) have been extensively reported. Other ASMs associated with alopecia were cenobamate (18), levetiracetam (14), topiramate (13), lacosamide (7), vigabatrin (6), phenobarbital (5), gabapentin (5), phenytoin (4), pregabalin (4), eslicarbazepine (3), brivaracetam (2), clobazam (2), perampanel (2), trimethadione (2), rufinamide (2), zonisamide (2), primidone (1), and tiagabine (1). There were no reports of oxcarbazepine and felbamate with drug-induced alopecia. Hair loss seen with ASMs was diffuse and non-scarring. Telogen effluvium was the most common cause of alopecia. A characteristic feature was the reversibility of alopecia after ASM dose adjustment. **Conclusions:** Alopecia should be considered one important adverse effect of ASMs. Patients reporting hair loss with ASM therapy should be further investigated, and specialist consultation is recommended.

## 1. Introduction

The mainstay of treatment for epilepsy is antiseizure medications (ASMs). Approximately 70% of individuals with epilepsy obtain seizure freedom with adequate ASMs therapy [1]. In this context, low literacy levels, high cost, availability, low tolerability, and irregular medication use are some factors related to non-compliance [2]. However, the adverse effects of ASMs remain one of the leading causes of non-adherence. Furthermore, adverse effects can significantly impact the quality of life in people with epilepsy [3]. Cosmetic side effects (CSEs) are the fourth most commonly reported side effect associated with ASMs [4]. Increased body weight, acne vulgaris, hirsutism, and alopecia are some CSEs already reported with ASMs. In a study with 1903 individuals taking commonly used ASMs, weight gain and hair loss were the most common causes of ASMs discontinuation [5]. Additionally, CSEs are estimated to cost around USD 3500 per patient due to therapeutical modifications, consultation costs, and treatment of adverse drug reactions [6]. In addition to the economic burden, CSEs can negatively impact social functioning and emotional well-being and decrease self-esteem and quality of life in epilepsy patients [4].

Alopecia is generally differentiated into cicatricial or scarring alopecia, nonscarring alopecia, and structural hair disorders. In non-scarring alopecia, hair follicle damage is not permanent [7,8]. Non-scarring alopecia is further classified into focal, diffuse, and patterned hair loss [9]. This review will focus on diffuse non-scarring hair loss, the most common type of alopecia found in drug-induced hair loss [10]. The majority of the literature on drug-induced alopecia is related to chemotherapy. Studies assessing the quality of life in individuals undergoing chemotherapy revealed that alopecia ranked amongst the most troublesome side effects affecting body image and was described as distressing [11]. Interestingly, there are no differences between men and women concerning body image regarding the degree of alopecia. However, women’s psychological well-being was lower than men’s because the incidence of alopecia was higher in women [12].

Drug-induced alopecia usually presents as diffuse, non-scarring hair loss commonly reversible upon drug discontinuation [13,14]. Telogen effluvium and anagen effluvium are the two main types of hair loss secondary to medications [15,16]. Anagen effluvium is abrupt hair loss in the anagen phase secondary to impairment of the mitotic or metabolic activity of the hair follicle [17,18]. In this context, anti-neoplastic agents are the most commonly reported drugs associated with anagen effluvium [19,20]. Other medications (bismuth, levodopa, colchicine, and cyclosporine) were rarely reported to cause alopecia [21,22]. Some authors hypothesized that the hair loss probably occurred due to the abrupt cessation of mitotic activity, which can weaken the partially keratinized proximal portion of the hair shaft [23]. This may lead to narrowing and eventual breakage of the hair canal, which can cause complete failure of hair formation. Hair shedding usually begins within the first three weeks of the offending drug. Near complete hair loss is well-established after three months. Interestingly, the scalp is the most common location for hair loss due to its long anagen phase [24].

Telogen effluvium is a common cause of diffuse hair loss [25]. This condition is characterized by hair follicles entering their resting phase (telogen) and falling out too early. People with telogen effluvium can shed more than 100 hairs a day. It usually occurs three months after the introduction of the causative drug. Drugs related to telogen effluvium include anticonvulsants, oral retinoids, oral contraceptives, antithyroid drugs, hypolipidemic drugs, beta-blockers, captopril, and amphetamines [26].

It is believed that the most common antiseizure medications associated with hair loss are valproate and carbamazepine [27]. Additionally, therapies that might improve antiseizure medication-induced alopecia still need to be studied. This study aims to review the literature regarding alopecia and hair loss as secondary effects of ASMs.

## 2. Methods

### 2.1. Search Strategy

Primarily, we searched six databases to locate studies on ASM-induced alopecia published in electronic form. Excerpta Medica (Embase), Google Scholar, Latin American & Caribbean Health Sciences Literature (Lilacs), Medline, Scientific Electronic Library Online (Scielo), and Science Direct were searched. Search terms were “alopecia, hair loss, antiepileptic medication, antiseizure medication, epilepsy, and seizure”. During our secondary search [(alopecia) AND (ASM)], we searched for alopecia in 23 ASMs, of which 21 were associated with hair loss (Table 1).

### 2.2. Inclusion and Exclusion Criteria

Original articles, case reports, case series, letters to editors, and bulletins were included in this review with no language restriction. Google Translate services were used when non-English literature was beyond the authors’ proficiency (Portuguese, English, Spanish, French, and German) or when the abstract in English could not provide enough information. The authors independently screened the titles and abstracts of all papers from the initial search. Disagreements between the authors were resolved through discussion.

All articles discussing at least one ASM causing hair loss were included. Cases where the cause of hair loss was already known and either alopecia did not worsen or was unrelated to ASMs were excluded. Furthermore, cases not accessible by electronic methods, including after a formal request to the authors (by email), were excluded. Patients with more than one contributing factor to alopecia were evaluated using the Naranjo algorithm to estimate the probability of the event occurring.

### 2.3. Data Extraction

**Figure 1:**
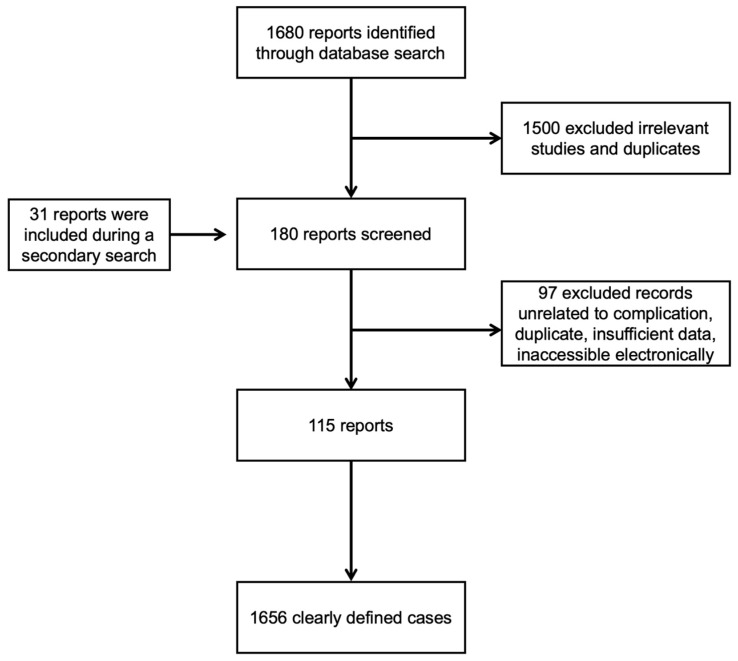
Flowchart of the screening process.

We identified 1680 articles from a primary search. We excluded 1500 articles based on the title and abstract. During our secondary investigation, we identified 151 articles. We included 31 articles from the secondary search. This review included 115 studies containing 1656 individuals (Figure 1).

## 3. Antiseizure Medications

### 3.1. Valproate (VPA)

VPA is one of the most frequently used ASMs for treating generalized and focal seizures. It is also indicated for managing bipolar disorders, neuropathic pain, and migraine prophylaxis [28]. VPA is associated with neurological and cosmetic side effects [29]. Alopecia is among the 10 most commonly reported adverse effects of VPA use [30]. The incidence of alopecia secondary to VPA greatly varies from 0.5 to 24% [31]. The diagnosis is based on the history of hair loss or abnormalities following VPA administration. Additionally, it can be confirmed by performing pull tests and modified wash tests.

Hair loss most commonly occurs after three to six months of VPA introduction [32]. Apparently, there is a direct relationship between the occurrence of hair loss and blood levels of VPA. High VPA blood levels (80–150 mcg/L) are associated with alopecia occurrence in 28% of the individuals taking VPA. On the other hand, adequate blood level concentrations (25–50 mcg/L) of VPA are related to a 4 percent occurrence of alopecia [33]. However, a meta-analysis found no significant correlation between the dose or duration of VPA therapy and alopecia [34].

**Figure 2:**
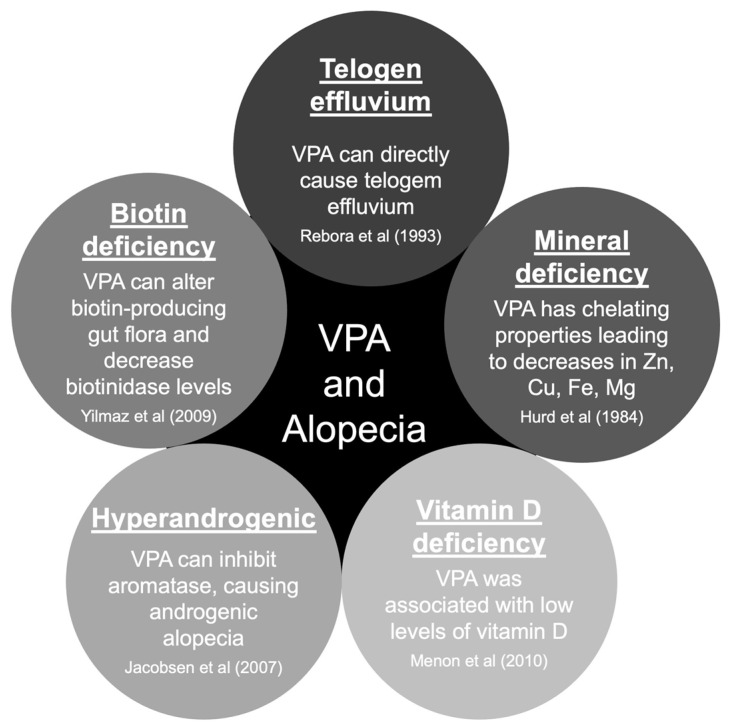
Pathophysiology of valproate-induced alopecia. Abbreviations: Cu, copper; Mg, magnesium; VPA, valproate/valproic acid; Zn, zinc [37,38,39,40,41].

There are five main hypotheses to explain hair loss associated with VPA use [35,36]. The pathophysiology of hair loss includes biotin deficiency, hyperandrogenism, mineral deficiency, telogen effluvium, and vitamin D deficiency (Figure 2) [37,38,39,40,41]. The hair loss associated with VPA is typically incomplete and usually reversible after discontinuing the causative drug [23]. Treatment will include general measures, such as reassurance and hair care techniques. Additionally, some authors reported specific treatment options that should be carefully evaluated case-by-case (Table 2) [35,36,42,43,44,45,46,47,48,49].

### 3.2. Carbamazepine (CBZ)

CBZ has a broad use for the treatment of focal seizures. It is also used for treating bipolar disorder and trigeminal neuralgia. Although CBZ is not as notorious for causing alopecia as VPA, the incidence of alopecia with it ranges from 0.3% to 6% [4,50]. In 1985, Shuper et al. reported the first case of CBZ-induced alopecia. An 8.5-year-old girl with headaches and multifocal epilepsy was managed with CBZ. Hair loss stopped after CBZ discontinuation, and hair regrowth was observed [51].

Pillans and Woods reported 177 cases of CBZ-induced alopecia in 1995 [33]. In 1992, Mattson et al. conducted a double-blind trial in 480 individuals with focal seizures and generalized tonic-clonic seizures using VPA or CBZ. Hair loss or abnormal hair texture was observed in 12 percent of subjects taking VPA and 6 percent taking CBZ [52].

In 1997, Ikeda et al. published two individuals that developed hair loss secondary to CBZ therapy. In the first case, the patient was treated for focal epilepsy. Hair loss developed after three months of starting the drug. The blood concentration of CBZ was maintained at 5.0 mg/L. Hair regrowth after replacing the offending drug was observed. The second case reported by Ikeda et al. was an individual diagnosed with tuberous sclerosis with focal seizures. CBZ was started and maintained at a steady level with a blood concentration of 6.5 mg/L. Hair loss was observed after two months of starting CBZ therapy. After reducing the CBZ dose from 600 mg/day to 200 mg/day, hair shedding was reduced, and new hair began to grow [53]. Another case report from Oh et al. demonstrated hair loss four months after starting CBZ at 600 mg/day. The hair loss was reversed after decreasing the dose of CBZ to 200 mg/day [54].

### 3.3. Lamotrigine (LTG)

LTG is used to treat epilepsy as a monotherapy or as an adjunct to other antiseizure polytherapy. It is a first-line treatment for primary generalized tonic–clonic seizures, focal seizures, atypical absence seizures, myoclonic seizures, and atonic seizures [55]. Apart from using it as an ASM, it can also be used in mood disorders and depression management [56]. The incidence of hair loss associated with LTG is 0.8%. Interestingly, three in every four individuals who developed LTG-induced alopecia reported this side effect as a significant factor for LTG withdrawal [4].

LTG-induced epidermal necrolysis with some hair loss is well known. However, there are few reports of isolated hair loss without epidermal necrolysis associated with LTG in the literature [57]. In 2004, Patrizi et al. reported the first case of LTG-induced alopecia. A 24-year-old female with focal epilepsy was started on LTG and magnesium VPA. The magnesium VPA dose was decreased to 600 mg/day, and the LTG dosage was increased to 100 mg/day. After a few months, hair loss was observed. Trichogram ruled out androgenetic alopecia. LTG probably was the main cause for the development of alopecia, but the effect of VPA on hair structure cannot be excluded [58].

In 2006, Hillemacher et al. reported a case of a 63-year-old diagnosed with bipolar disorder. LTG was started and increased to 150 mg/day. Hair loss was noted in the third week of treatment. A trichogram showed hair with a prolonged telogen phase and dystrophic characteristics [59]. This strongly suggested telogen effluvium as the cause of LTG-induced alopecia. In 2017, Solmi et al. reported a patient affected by treatment-resistant major depressive disorder who was managed with LTG, and telogen effluvium was observed [60].

Tengstrand et al.’s study is one of the most significant articles in the literature regarding LTG-induced alopecia. The authors assessed 337 individuals from 19 countries who developed hair loss associated with LTG. In 291 reports, alopecia developed with LTG monotherapy. They found that alopecia should be considered a significant factor affecting adherence to LTG therapy. The time to onset of alopecia after LTG intake was variable, in which 17 individuals presented alopecia within one month, 48 presented between one and six months, and 31 presented after six months of LTG onset [61]. It is worth mentioning that Tengstrand et al.’s study assessed VigiBase reports, a database with limited information on patient demographic characteristics and clinical descriptions [61].

### 3.4. Levetiracetam (LEV)

LEV is considered a broad-spectrum ASM. This drug was approved by the FDA in 1999 for the management of epilepsy [62]. Interestingly, LEV differs in structure and mechanism of action from other marketed ASMs [63]. LEV has favorable pharmacokinetics and a low potential for drug interactions [64]. In a study with 1903 individuals, a 0.4% prevalence of hair loss due to LEV therapy was observed [4].

Hair loss is a rare adverse effect of LEV therapy. In 2014, Zou et al. reported five cases of LEV-induced alopecia. The doses of LEV ranged within 500–1000 mg/day. Hair loss secondary to LEV was observed to occur between three and eight weeks of LEV therapy. The individuals presented with diffuse non-scarring hair loss. Complete recovery from alopecia was seen in two out of five subjects. The other two patients noticed an improvement in hair loss after decreasing the dose from 1000 mg/day to 750 mg/day. One individual decided to continue with medication despite hair loss [65]. The authors concluded that the alopecia associated with LEV was due to telogen effluvium. Aghamollaii et al. published a case series of three patients that experienced hair loss with LEV [66].

### 3.5. Gabapentin (GBP)

GBP is associated with mild adverse events, such as somnolence, fatigue, ataxia, and dizziness, which are reported in about three in every four patients [67]. It is a first-line treatment for the management of neuropathic pain [68]. Eker et al. reported a case of alopecia with GBP therapy for neuropathic pain. The patient was started on GBP 1800 mg/day. Hair loss was noticed one week after the GBP therapy onset. Patchy areas of alopecia were noticed. When GBP was discontinued, hair regrowth was observed [69].

The first case of GBP-induced alopecia was reported in 1997 by Picard et al. A 15-year-old girl with seizures was taking CBZ and phenytoin. GBP 1800 mg/day was prescribed as add-on therapy. The patient experienced alopecia during the second month of treatment with GBP. Hair loss was diffuse without bald patches. GBP was discontinued, but CBZ and phenytoin were continued. Hair regrowth was observed three weeks after GBP discontinuation, and alopecia was completely reversed within one month [70].

### 3.6. Topiramate (TPM)

TPM may cause alopecia in approximately one percent of its users [8]. According to Chen et al., alopecia prevalence was 1.7% among 230 TPM users. TPM was discontinued in all the individuals who developed alopecia because the patients decided to stop the treatment [4]. Chuang et al. reported a 15-year-old girl with frontal lobe epilepsy who developed hair loss after two months of TPM adjunctive therapy. The hair loss was reversible upon discontinuation of the drug. There was a recurrence of alopecia after the TPM rechallenge [71]. Another case report demonstrated hair loss that started after three months of starting TPM at a dosage of 50 mg/day for migraine headaches. Hair started regrowing with a dose reduction to 25 mg/day, and alopecia recurred after the reintroduction of a 50 mg/day dosage of TPM [72].

Lagrand et al. described a case of an individual who developed alopecia with different medications for managing her tremor-dominant Parkinson’s disease. She presented hair loss after the introduction, in different moments, of levodopa/benserazide, propranolol, and TPM [73]. Their case is interesting because it suggests a possible genetic predisposition for the development of alopecia after the administration of determined groups of medications.

### 3.7. Phenytoin (PHT)

As part of the Columbia and Yale ASM Database Project, Chen et al. reviewed patient records, including demographics, medical history, ASM use, and side effects for 1903 adult patients (≥16 years of age) newly started on an ASM. Cosmetic side effects were determined by patient or physician reports in the medical record, including acne, gingival hyperplasia, hair loss, hirsutism, and weight gain. PHT was taken by 404 out of 1903 patients. In total, 0.3% attributed hair loss due to PHT and 0.3% intolerability due to hair loss [4]. Herranz et al. assessed the clinical side effects of long-term monotherapy of phenobarbital, primidone, PHT, CBZ, and VPA in 392 pediatric individuals. PHT was more commonly associated with cosmetic side effects than the other investigated drugs. However, there was no report of PHT-induced alopecia. Instead, hirsutism was observed in 9% of children taking PHT [74]. Interestingly, VPA was the drug most commonly associated with alopecia, which occurred in 0.8% of children [75].

Kuhne et al. described a case of Munchausen by proxy syndrome mimicking childhood-onset systemic lupus erythematosus. A patient presenting with malar rash, photosensitivity, alopecia, arthralgia, arterial hypertension, macroscopic hematuria, seizure, and positive antinuclear antibodies was reported. The pediatric patient received high doses of PHT, which led to drug-induced lupus erythematosus [76]. Thus, subjects presenting with alopecia during PHT therapy should be assessed for other clinical manifestations suggesting autoimmune diseases. There are two other cases in the literature with alopecia secondary to PHT-induced lupus erythematosus [77,78].

Hirsutism is more frequent than alopecia as an adverse effect of PHT [75]. In this context, a study assessed the effectiveness of PHT in suppressing chemotherapy-induced hair loss. The authors revealed that oral PHT could significantly suppress hair loss due to cyclophosphamide therapy in rats. PHT co-administration was related to improved hair growth, increased hair-shaft thickness, and reduced skin lipid peroxidation [79].

### 3.8. Pregabalin (PGB)

Isolated alopecia secondary to PGB was uncommonly described in the literature. Chen et al. found only one patient who developed alopecia among 143 PGB users [4]. In another study with the Netherlands Pharmacovigilance Centre Lareb data, the incidence of PGB-induced alopecia was 0.07% [80]. Noteworthily, PGB dose may be directly associated with hair loss. In Wistar rats, alopecia was more frequently observed with higher doses of PGB [81].

Turgut et al. reported an adult female diagnosed with fibromyalgia. PGB 75 mg/day was started, and the dose was increased to 150 mg/day after one week. Within three weeks of PGB therapy, significant hair loss was observed. PGB was discontinued. Complete hair regrowth was observed after two weeks of PGB withdrawal [82].

In clinical practice, PGB-induced alopecia is commonly seen as part of drug reactions with eosinophilia and systemic symptoms (DRESS). However, it was scarcely reported in the literature. Suh et al. described an individual presenting with alopecia and pruritic erythema after PGB use. DRESS diagnosis was made. After PGB discontinuation, hair regrowth was observed [83].

### 3.9. Perampanel (PMP)

PMP has been approved as an add-on treatment for refractory focal seizures and primary generalized tonic–clonic seizures in idiopathic generalized epilepsy [84]. The most frequently reported side effects are dizziness and fatigue. In this context, the only described CSE were weight gain (7.4–19.2%) and skin rash (10.6%) [85,86,87]. It is worth mentioning that these CSEs were only observed in specific populations, such as Asian individuals [87].

Rohracher et al. pooled observational data of PMP comprising a full analysis set of 2396 individuals. Only one individual reported alopecia during the first year of PMP therapy [88]. Villanueva et al. assessed the safety and efficacy of long-term PMP in 464 subjects. The incidence of PMP-induced alopecia was 0.2% [89].

The mechanism of action of PMP involves a non-competitive antagonism of the α-amino-3-hydroxy-5-methyl-4-isoxazolepropionic acid (AMPA) receptor. The AMPA receptors are considered the major subtype of ionotropic glutamate receptors [90]. Noteworthy glutamate can promote hair growth in mice models. Additionally, it is believed that glutamic acid can control hair follicle proliferation, decreasing gene expression related to apoptosis in the skin and increasing cell viability and proliferation in human keratinocytes [91]. Therefore, the low levels of glutamate in PMP therapy may be associated with hair loss.

### 3.10. Phenobarbital, Vigabatrin, Tiagabine, and Trimethadione

Phenobarbital-induced isolated alopecia was rarely described. Alopecia was usually described as part of an anticonvulsant hypersensitivity syndrome induced by phenobarbital. Huang et al. reported an individual presenting with alopecia in the convalescent status of phenobarbital-induced anticonvulsant hypersensitivity syndrome. Skin histology revealed peri-follicular, peri-bulbar, and supra-bulbar lymphocyte infiltration [92]. In the literature, two other cases of alopecia were present in the convalescent period of phenobarbital hypersensitivity syndrome [93,94]. All individuals reported they had a favorable prognosis with complete hair regrowth within three months [92,93,94]. Huang et al. proposed that phenobarbital could promote lymphocyte infiltration into the peri-follicular, peri-bulbar, and supra-bulbar anatomical regions. Additionally, phenobarbital dose may be associated with the incidence of alopecia. Ghorani-Azam et al. systematically reviewed the literature concerning phenobarbital in neonates and pediatrics that were treated for seizures. Interestingly, alopecia was a reported sign of phenobarbital poisoning [95].

Vigabatrin has also been reported to cause hair loss. In Lampl et al., 5 out of 52 patients who received vigabatrin for focal seizures experienced moderate hair loss or changes in hair structure. The complaints began after three to seven weeks of initiating vigabatrin treatment. Hair loss recovery was seen in all patients after cessation of treatment [96]. Vigabatrin showed different results in rodent studies. It was observed that mice tolerated high doses of vigabatrin, but Sprague Dawley rats developed acute alopecia and body weight gain. These CSEs were improved within six months of vigabatrin discontinuation [97].

Tiagabine may increase the level of γ-aminobutyric acid. Vossler et al. assessed the adverse effects of long-term tiagabine use in 292 subjects. Only one patient developed alopecia secondary to tiagabine [98]. Another study by Mercke et al. found an incidence of 1% of tiagabine-induced alopecia [8].

Trimethadione is historically important for managing absence seizures and refractory temporal lobe epilepsy. There are some case reports in the literature about alopecia associated with this medication [99].

For further description of the cases reported in the literature about alopecia secondary to antiseizure medication, refer to Table 3. Table 4 shows the results from PubMed, cases encountered in the literature, and incidence of ASM-induced alopecia.

## 4. Discussion

VPA, CBZ, and LTG were the most studied causes of ASM-induced alopecia. Additionally, LEV, TPM, and cenobamate were uncommonly associated with hair loss. However, the rest of the anticonvulsants were rarely reported with alopecia in the literature. To the author’s knowledge, there were no cases of oxcarbazepine and felbamate affecting hair growth. Interestingly, alopecia was usually seen with higher than the usual therapeutic doses of many ASMs [66]. However, there is no established dose-dependent relationship, and in a few cases, hair loss occurred at lower doses of ASM [106].

In most cases reported, hair loss stopped after dose adjustment of the ASM, which included discontinuing the drug or decreasing its dose [70]. In many cases, hair loss recured after ASM reintroduction or an increased dose of ASM back to the original strength that caused alopecia in the first place [72]. Nevertheless, the reversibility of alopecia has been seen as a characteristic feature in all ASMs [73]. Noteworthily, the optimal therapeutic dosage of antiseizure medications should be carefully assessed, especially in individuals with multiple comorbidities due to the modified pharmacokinetics and pharmacodynamics of drugs, which can lead to high percentages of adverse events [172].

The time duration has also been variable in the examples mentioned in this review. The most common duration from starting the drug or increasing the dosage to the onset of hair loss was between one and six months. However, it can vary with the different drugs and their doses. We have mentioned different timelines and associated doses at which hair loss was reported in Table 2. Moreover, hair loss etiology probably was related to telogen effluvium due to features such as non-scarring diffuse distribution, time of alopecia onset, and reversible nature of hair loss. Other than one case, anagen effluvium was not associated with the main cause of ASM-induced alopecia [116].

Telogen effluvium can be diagnosed by identifying known triggers from the history in the three to four months preceding the onset of hair shedding. Additionally, endocrine, nutritional, and autoimmune etiologies should be ruled out. Usually, the diagnosis of telogen effluvium was made by demonstrating a compatible chronology of ASM exposure and onset of hair loss. Hair regrowth within three months of ASM discontinuation can support diagnosis. A drug rechallenge was attempted in some individuals with a re-occurrence of hair loss [43].

Further tests to diagnose telogen effluvium are a hair pull test, 60-second timed hair count, trichogram, trichoscopy, and scalp biopsy [173]. A scalp biopsy can be conducted to identify the earliest stages of androgenetic alopecia, which was already reported with VPA [163]. However, in almost all the studies we analyzed, history was sufficient to label telogen effluvium [174]. A trichogram was performed in only a few reported cases [115,116].

The hair pull test is strongly positive for telogen effluvium [175]. It is carried out by grasping 40–60 closely grouped scalp hair with the thumb and index finger, and gentle traction is applied as the hair is pulled firmly and slowly from the scalp. Normally, only two to three hairs come out. In telogen effluvium, more than 10 percent of hair is easily pulled out from any part of the scalp [176].

In telogen effluvium, the average number of scalp hairs shed daily ranges from 30 to 100, although there is a seasonal fluctuation with the greatest loss around late summer [177,178]. Inspection of telogen hairs with the naked eye can distinguish anagen from telogen hairs [179]. Telogen hairs have depigmented hair bulbs and the absence of inner root sheaths, whereas anagen hairs have inner root sheaths [180]. The inner root sheath can be subdivided into three layers, the cuticle, Huxley’s, and outer Henle’s layers.

Trichogram can help in telogen effluvium diagnosis. Trichogram from a hair pluck sample usually shows more than 25 percent telogen hair in acute telogen effluvium [181]. A 60-s timed hair count can also be performed. Usually, more than 100 hairs will be observed with telogen effluvium (the normal value is 10 hairs) [182]. Another method, which involves combing the air forward for 60 s over a contrasting cloth before shampooing, can be used to assess the disease progression and resolution [183].

An interesting association between hair loss and ASMs is blood zinc concentrations. Low blood zinc levels have been reported in association with higher levels of LEV, especially in cases with reported hair loss [66]. In their letter to the editor, Calabro et al. discussed the relationship between LEV and blood zinc levels. They described a case of a 35-year-old female who was started on LEV and titrated up to 1500 mg/day for generalized tonic–clonic seizures. In the second month’s follow-up, she noticed patchy alopecia. Her zinc levels were below normal (45 μg/dL). Six weeks after LEV discontinuation, hair regrowth was noted. At a one-year follow-up, serum zinc levels were back to normal [106]. Another reported case showed borderline low normal zinc levels one year after stopping LEV due to LEV-induced alopecia [107]. Zou et al. reported that zinc supplementation effectively treated LEV-induced alopecia in five individuals [65]. In this context, Aghamollai et al. prescribed zinc sulfate supplementation for managing hair loss due to LEV. The authors described three patients with alopecia secondary to LEV for whom zinc sulfate 220 mg/day was started. In all three cases, hair loss improvement was seen [66].

VPA-induced alopecia has also been linked with abnormal concentrations of zinc. It has been suggested that low zinc levels may occur due to VPA-induced zinc chelation [184]. However, evidence has shown variable results of zinc blood levels in VPA-treated patients [8]. In animal models, VPA has been shown to induce zinc and selenium deficiencies [44,185,186]. Suzuki et al. showed that long-term ASM therapy, including VPA and CBZ, can decrease zinc levels in the male sex. Interestingly, increased copper concentrations were found in the female sex with long-term ASM therapy [187]. In this context, Kuzuya et al. demonstrated no change in serum zinc or copper levels in patients with epilepsy who had undergone one month of VPA therapy [188]. Therefore, we hypothesize that ASMs can lead to abnormal concentrations of some metals by influencing the absorption of these substances. This can partially explain the development of abnormal levels of zinc and copper with chronic instead of acute ASM therapy.

Zinc and selenium supplementations were already prescribed for VPA-induced alopecia. Fatemi et al. reported an individual with refractory bipolar disorder who developed alopecia secondary to VPA, leading to drug discontinuation. A VPA therapy rechallenge was attempted with zinc and selenium supplementation. There was no occurrence of any hair structural abnormality [44]. Asadi–Pooya systematically reviewed the literature regarding the cosmetic adverse effects of antiseizure medications. They found strong evidence of ASM associated with cosmetic adverse effects. PHT was extensively reported to cause gingival hyperplasia, hirsutism, and acne. VPA was related to causing hair loss and hirsutism. Additionally, they suggest that the evidence for zinc or biotin supplementation in VPA and LEV-associated alopecia needs to be further assessed [189].

There are important limitations in the present study that should be discussed. First, the data in the literature regarding alopecia secondary to antiseizure medication are diverse and sometimes even contradictory. Second, different types of studies with different outcomes to provide an overall view of the literature were included. Third, a significant number of reports only observed alopecia as a secondary outcome, which can affect the incidence of alopecia throughout the studies. Fourth, the majority of the reports of management were about valproate-induced alopecia, but these reports were isolated and had a low level of evidence.

## 5. Future Perspectives

Future studies with ASMs should assess hair abnormalities with a trichogram. Observing hair structures is essential for diagnosing telogen effluvium. Special attention should be paid to those ASMs whose hair abnormalities were already noted in animal studies. Additionally, the complaints by the individuals in clinical trials with ASM should be further assessed. A mild report of hair loss with ASM should be investigated, and the patient should be recommended to a specialist. Moreover, clinical trials with the supplementation of metal and vitamins in ASM-induced alopecia are important. The development of strong evidence with different treatment options can improve the quality of life and management in people with epilepsy, mainly in those seizure-free individuals with specific types of medications, in which a small dose adjustment can lead to the development of new seizures.

Interestingly, most reports in the literature only described hair loss as a secondary objective of antiseizure medication. Noteworthily, only primary objectives are considered significant due to statistical power. Therefore, future studies need to investigate alopecia as a primary objective of hair loss for further epidemiological assessment and causality. Moreover, most studies describing the management of ASM-induced alopecia are case reports, which can significantly impact the evidence of therapeutic choice.

Understanding ASM’s interaction with genes to cause adverse effects is also important. Pharmacogenomic studies can provide crucial information regarding the potential adverse reactions that the patient may develop with the use of specific medication. These studies are mandatory in individuals with ASM-induced alopecia because there is a significantly higher incidence of VPA-induced alopecia in European and American populations when compared to Asian individuals.

## 6. Conclusions

Drug-related hair loss is challenging to diagnose. It requires an understanding of normal hair growth and the different causal factors that are involved in it. In this context, hair loss is an important adverse effect of ASM because it affects a significant percentage of the population. Additionally, it can negatively impact the quality of life of people with epilepsy leading to poor therapeutical adherence and a high economic burden. VPA, LEV, and LTG-associated alopecia were extensively reported in the literature. Management of alopecia includes reassurance, hair care techniques, and drug modification. The most frequent management was ASM discontinuation. In some cases, ASM dose reduction was attempted. Hair loss secondary to ASMs was reversible. Metals and vitamin supplementations in the management of ASM-induced alopecia should be investigated.

## Figures and Tables

**Table 1:** FreeText and MeSH search terms in the US National Library of Medicine.

Query	MeSH Terms	Results
(alopecia) AND (valproate)	(“alopecia”[MeSH Terms] OR “alopecia”[All Fields] OR “alopecias”[All Fields]) AND (“valproat”[All Fields] OR “valproate s”[All Fields] OR “valproates”[All Fields] OR “valproic acid”[MeSH Terms] OR (“valproic”[All Fields] AND “acid”[All Fields]) OR “valproic acid”[All Fields] OR “valproate”[All Fields])	70
(alopecia) AND (carbamazepine)	(“alopecia”[MeSH Terms] OR “alopecia”[All Fields] OR “alopecias”[All Fields]) AND (“carbamazepine”[MeSH Terms] OR “carbamazepine”[All Fields] OR “carbamazepin”[All Fields] OR “carbamazepines”[All Fields] OR “carbamazepine s”[All Fields])	31
(alopecia) AND (phenytoin)	(“alopecia”[MeSH Terms] OR “alopecia”[All Fields] OR “alopecias”[All Fields]) AND (“phenytoin”[MeSH Terms] OR “phenytoin”[All Fields] OR “phenytoine”[All Fields] OR “phenytoin s”[All Fields] OR “phenytoins”[All Fields])	20
(alopecia) AND (phenobarbital)	(“alopecia”[MeSH Terms] OR “alopecia”[All Fields] OR “alopecias”[All Fields]) AND (“phenobarbital”[MeSH Terms] OR “phenobarbital”[All Fields] OR “phenobarbitals”[All Fields])	16
(alopecia) AND (lamotrigine)	(“alopecia”[MeSH Terms] OR “alopecia”[All Fields] OR “alopecias”[All Fields]) AND (“lamotrigin”[All Fields] OR “lamotrigine”[MeSH Terms] OR “lamotrigine”[All Fields] OR “lamotrigine s”[All Fields])	10
(alopecia) AND (levetiracetam)	(“alopecia”[MeSH Terms] OR “alopecia”[All Fields] OR “alopecias”[All Fields]) AND (“levetiracetam”[MeSH Terms] OR “levetiracetam”[All Fields])	8
(alopecia) AND (gabapentin)	(“alopecia”[MeSH Terms] OR “alopecia”[All Fields] OR “alopecias”[All Fields]) AND (“gabapentin”[MeSH Terms] OR “gabapentin”[All Fields] OR “gabapentine”[All Fields] OR “gabapentin s”[All Fields])	6
(alopecia) AND (topiramate)	(“alopecia”[MeSH Terms] OR “alopecia”[All Fields] OR “alopecias”[All Fields]) AND (“topiramate”[MeSH Terms] OR “topiramate”[All Fields] OR “topiramate s”[All Fields])	5
(alopecia) AND (oxcarbazepine)	(“alopecia”[MeSH Terms] OR “alopecia”[All Fields] OR “alopecias”[All Fields]) AND (“oxcarbazepin”[All Fields] OR “oxcarbazepine”[MeSH Terms] OR “oxcarbazepine”[All Fields])	4
(alopecia) AND (clobazam)	(“alopecia”[MeSH Terms] OR “alopecia”[All Fields] OR “alopecias”[All Fields]) AND (“clobazam”[MeSH Terms] OR “clobazam”[All Fields])	2
(alopecia) AND (felbamate)	(“alopecia”[MeSH Terms] OR “alopecia”[All Fields] OR “alopecias”[All Fields]) AND (“felbamate”[MeSH Terms] OR “felbamate”[All Fields])	2
(alopecia) AND (vigabatrin)	(“alopecia”[MeSH Terms] OR “alopecia”[All Fields] OR “alopecias”[All Fields]) AND (“vigabatrin”[MeSH Terms] OR “vigabatrin”[All Fields] OR “vigabatrine”[All Fields])	2
(alopecia) AND (pregabalin)	(“alopecia”[MeSH Terms] OR “alopecia”[All Fields] OR “alopecias”[All Fields]) AND (“pregabalin”[MeSH Terms] OR “pregabalin”[All Fields] OR “pregabalin s”[All Fields] OR “pregabaline”[All Fields])	1
(alopecia) AND (primidone)	(“alopecia”[MeSH Terms] OR “alopecia”[All Fields] OR “alopecias”[All Fields]) AND (“primidone”[MeSH Terms] OR “primidone”[All Fields] OR “primidon”[All Fields])	1
(alopecia) AND (trimethadione)	(“alopecia”[MeSH Terms] OR “alopecia”[All Fields] OR “alopecias”[All Fields]) AND (“trimethadione”[MeSH Terms] OR “trimethadione”[All Fields])	1
(alopecia) AND (zonisamide)	(“alopecia”[MeSH Terms] OR “alopecia”[All Fields] OR “alopecias”[All Fields]) AND (“zonisamid”[All Fields] OR “zonisamide”[MeSH Terms] OR “zonisamide”[All Fields] OR “zonisamide s”[All Fields])	1
(alopecia) AND (brivaracetam)	(“alopecia”[MeSH Terms] OR “alopecia”[All Fields] OR “alopecias”[All Fields]) AND (“brivaracetam”[Supplementary Concept] OR “brivaracetam”[All Fields])	0
(alopecia) AND (cenobamate)	(“alopecia”[MeSH Terms] OR “alopecia”[All Fields] OR “alopecias”[All Fields]) AND (“cenobamate”[Supplementary Concept] OR “cenobamate”[All Fields] OR “cenobamate”[All Fields])	0
(alopecia) AND (eslicarbazepine)	(“alopecia”[MeSH Terms] OR “alopecia”[All Fields] OR “alopecias”[All Fields]) AND (“eslicarbazepine”[Supplementary Concept] OR “eslicarbazepine”[All Fields])	0
(alopecia) AND (lacosamide)	(“alopecia”[MeSH Terms] OR “alopecia”[All Fields] OR “alopecias”[All Fields]) AND (“lacosamide”[MeSH Terms] OR “lacosamide”[All Fields])	0
(alopecia) AND (perampanel)	(“alopecia”[MeSH Terms] OR “alopecia”[All Fields] OR “alopecias”[All Fields]) AND (“perampanel”[Supplementary Concept] OR “perampanel”[All Fields])	0
(alopecia) AND (rufinamide)	(“alopecia”[MeSH Terms] OR “alopecia”[All Fields] OR “alopecias”[All Fields]) AND (“rufinamide”[Supplementary Concept] OR “rufinamide”[All Fields])	0
(alopecia) AND (tiagabine)	(“alopecia”[MeSH Terms] OR “alopecia”[All Fields] OR “alopecias”[All Fields]) AND (“tiagabine”[MeSH Terms] OR “tiagabine”[All Fields])	0

**Table 2:** Management of VPA-associated alopecia by Praharaj et al. [36] adapted by Rissardo et al.

Management	Comment	Reference
General measures	Reassurance. Alopecia is a benign side effect and is usually reversible. Additionally, provide hair care techniques. Advise to use soft brushes and mild shampoos and avoid dyes, heated curlers, and hair dryers.	Praharaj et al. (2022) [36]
Adjustment of VPA dosage	If feasible, VPA should be discontinued. Dose reduction of VPA was associated with hair regrowth. Additionally, a gradual increase in VPA dose was effective in the management of neurological conditions without affecting hair growth in some cases.	Uehlinger et al. (1992) [42] Henriksen et al. (1982) [43] Wang et al. (2019) [35]
Mineral supplementation	Iron, copper, magnesium, selenium, and zinc could be useful in treating hair loss associated with VPA. Zinc and selenium supplementation can help prevent further hair loss and promote regrowth.	Fatemi et al. (1995) [44] Trost et al. (2006) [45]
Vitamin supplementation	Oral administration of biotin (10 mg/day) shorted hair regrowth time. Other vitamins can be prescribed, but there is no evidence of their efficacy.	Castro-Gago et al. (2010) [46] Yilmaz et al. (2009) [38]
Agomelatine administration	Agomelatine was associated with a reduction of hair loss related to VPA. Agomelatine use should be attempted when the offending drug cannot be discontinued.	Sahin et al. (2017) [47]
Minoxidil administration	Minoxidil was associated with a reduction of hair loss related to VPA. Minoxidil use should be attempted when the offending drug cannot be discontinued.	Thomson et al. (2017) [48]
Topical VPA therapy	Topical application of VPA was found to promote hair growth.	Kakunje et al. (2018) [49]
Others	Advise the patient not to take VPA during meals to reduce its chelating effect on metals. VPA can affect zinc and selenium absorption, two metals associated with hair growth.	Praharaj et al. (2022) [36]

Abbreviations: VPA, valproate/valproic acid.

**Table 3:** Literature review of antiseizure medication-induced alopecia.

Case Reports									
Reference	ASM	Diagnosis	Dose (mg/day)	Duration ^a^		Comment ^b,c^			
Shuper et al. (1985) [51]	CBZ	Epilepsy	150–300	1 month		Hair loss reversed when CBZ was discontinued. The patient was also taking propranolol, known to cause hair shedding. However, propranolol was discontinued six months before the initiation of CBZ. Objective: primary objective. GRADE: Very low.			
Wadhwa et al. (1997) [100]	CBZ	Epilepsy	NA	NA		CBZ-hypersensitivity syndrome presenting with alopecia. Objective: primary objective. GRADE: Very low.			
Kohno et al. (2004) [101]	CBZ	NA	NA	1 week		The serum concentration of CBZ was 8.6 mcg/mL (therapeutic range: 8–12 mcg/mL). CBZ was discontinued, and hair loss stopped. Hair regrowth was observed within several days. Objective: primary objective. GRADE: Very low.			
Oh et al. (2008) [54]	CBZ	Neuropathic pain	600	3 months		Alopecia was seen at CBZ 600 mg/day. The hair pull test was positive. Alopecia was reversed with a CBZ dose reduction to 200 mg/day. Objective: primary objective. GRADE: Very low.			
Zenkov et al. (2008) [102]	CBZ	Epilepsy	NA	Weeks (undefined)		CBZ was discontinued. Complete hair regrowth was observed within one year. Objective: primary objective. GRADE: Very low.			
Kenyon et al. (2014) [103]	CBZ	GTCS	NA	4.5 years		CBZ was replaced by OXC. Objective: primary objective. GRADE: Very low.			
Rathore et al. (2021) [104]	CBZ	Focal epilepsy	200	3 days		Hair regrowth was observed within two months of management. Objective: primary objective. GRADE: Very low.			
Picard et al. (1997) [70]	GBP	Focal epilepsy	1800	4–8 weeks		Hair regrowth commenced three weeks after GBP discontinuation. A complete reversal of alopecia was observed in the first month of follow-up. Objective: primary objective. GRADE: Very low.			
Eker et al. (2009) [69]	GBP	Neuropathic pain	1800	1 week		Significant hair loss with patchy areas of alopecia among areas of normal hair growth was seen. Hair shedding was more evident in the frontal and parietal regions. Shedding stopped two months after the discontinuation of GBP. Objective: primary objective. GRADE: Very low.			
Chen et al. (2010) [105]	GBP	Postherpetic neuralgia	1200	3 weeks		Hair regrowth started four weeks after GBP discontinuation. Her hair growth resumed completely within three months. Objective: primary objective. GRADE: Very low.			
Calabro et al. (2013) [106]	LEV	GTCS	1500	1 month		During LEV therapy, serum zinc levels were low (45 μg/dL [normal range: 70–150 μg/dL]). After one year of LEV discontinuation, serum zinc levels increased to 90 μg/dL. Objective: primary objective. GRADE: Very low.			
Hamd et al. (2018) [107]	LEV	Focal epilepsy	750	2 months		Hair loss showed moderate improvement after discontinuation of LEV. Additionally, zinc intake was advised. Objective: primary objective. GRADE: Very low.			
Missori et al. (2023) [108]	LEV	Epilepsy	1000	4 weeks		LEV was discontinued. The progression of alopecia stopped immediately, and the patient’s hair mostly grew back three months later. Objective: primary objective. GRADE: Very low.			
Patrizi et al. (2005) [58]	LTG	NA	100	Months		The exact duration was not specified. It is possibly the first case of alopecia secondary to LTG. Objective: primary objective. GRADE: Very low.			
Hillemacher et al. (2006) [59]	LTG	BD	150	3 weeks		Rapid regression of hair loss was seen after LTG discontinuation. Objective: primary objective. GRADE: Very low.			
Solmi et al. (2017) [60]	LTG	Major depressive disorder	NA	NA		Naranjo algorithm scored 8 (probable). Objective: primary objective. GRADE: Very low.			
Krivda et al. (2022) [109]	LTG	BD type II	100	5 weeks		LTG-associated hypersensitivity syndrome with development of extensive alopecia. Objective: primary objective. GRADE: Very low.			
Turgut et al. (2020) [82]	PGB	Fibromyalgia	150	21 days		PGB was discontinued. Hair regrowth was complete after two weeks. Objective: primary objective. GRADE: Very low.			
Suh et al. (2016) [83]	PGB	Postherpetic neuralgia	NA	36 weeks		She presented focal alopecia and pruritic erythema. After PGB discontinuation, hair regrowth was observed.			
Knutsen et al. (1986) [93]	PHB	Epilepsy	NA	3 months		Skin biopsy: prominent miniaturization, no terminal hair, and perifollicular–peribulbar inflammation. Alopecia outcome: hair regrowth within three months. Triamcinolone acetonide was started. Objective: primary objective. GRADE: Very low.			
Bavdekar et al. (2004) [94]	PHB	Epilepsy	NA	3 months		Skin biopsy: hyperkeratosis with follicular plugging and a prominent mononuclear perivascular and dermal infiltrate. A prominent panvascular mononuclear cell infiltrates the submucosa and migration to the epidermis, causing spongiosis and the presence of “mummified” cells. Alopecia outcome: hair regrowth was observed within two weeks. Prednisolone was started. Objective: primary objective. GRADE: Very low.			
Huang et al. (2009) [92]	PHB	Epilepsy	NA	13th day		Skin biopsy: not performed. Alopecia outcome: total recovery within two months. Objective: primary objective. GRADE: Very low.			
Mangalvedhekar et al. (2001) [77]	PHT	NA	NA	NA		PHT-induced lupus erythematous. The patient presented with alopecia. Objective: primary objective. GRADE: Very low.			
Neki et al. (2015) [78]	PHT	GTCS	300	2 years		PHT-induced lupus erythematous. The patient presented with alopecia. Objective: primary objective. GRADE: Very low.			
Kuhne et al. (2019) [76]	PHT	GTCS	NA	NA		The patient developed alopecia probably due to PHT-induced lupus erythematous. Objective: primary objective. GRADE: Very low.			
Chuang et al. (2002) [71]	TPM	Focal epilepsy	NA	2 months		Reversible alopecia upon discontinuation of TPM. Recurred after reintroduction of TPM. Objective: primary objective. GRADE: Very low.			
Ghafoor et al. (2017) [72]	TPM	Migraine	50	3 months		Alopecia stopped with the dose reduction to TPM 25 mg/day. Alopecia recurred with increasing the dose to 50 mg/day. Objective: primary objective. GRADE: Very low.			
Lagrand et al. (2021) [73]	TPM	Tremor-dominant Parkinson’s disease	50	2 weeks		Hair regrowth was observed some weeks after. In this patient, alopecia was observed with levodopa, propranolol, and TPM. Objective: primary objective. GRADE: Very low.			
Laljee et al. (1980) [110]	VPA	Epilepsy	NA	NA		Persistent hair loss was reported in one individual. Objective: primary objective. GRADE: Very low.			
Uehlinger et al. (1992) [42]	VPA	Schizophrenia	1300	3 months		The VPA was discontinued after alopecia. A rechallenge of VPA did not cause hair loss. Noteworthily, the VPA reintroduction was titrated. Objective: primary objective. GRADE: Very low.			
Fatemi et al. (1995) [44]	VPA	BD	3000	NA		Zinc supplementation led to alopecia remittance. Transient return of alopecia, which was remitted after a temporary VPA dose reduction. Objective: primary objective. GRADE: Very low.			
McKinney et al. (1996) [28]	VPA	BD	1500	6 weeks		Multivitamin supplementation with selenium was started, but hair loss continued. VPA dose was reduced to 1000 mg/day and maintained. After 16 weeks, hair loss slowed. On the fifth month of VPA therapy, hair regrowth was observed. Objective: primary objective. GRADE: Very low.			
Khan et al. (1999) [111]	VPA	BD, epilepsy	NA	NA		A patient who developed acute transient alopecia of all scalp hair after a suicidal attempt with VPA overdose. Objective: primary objective. GRADE: Very low.			
Cinbis et al. (2007) [112]	VPA	NA	NA	7th day		Alopecia was a secondary effect of a low dose of VPA. The pediatric individual was taking VPA 10 mg/kg/day. Objective: primary objective. GRADE: Very low.			
Wilting et al. (2007) [113]	VPA	NA	1000	NA		Hair texture changes were noted with increasing the dose of VPA from 1000 to 2000 mg/day. When the dosage was lowered again to 1000 mg/day, curliness disappeared but hair thinning was still present. Objective: primary objective. GRADE: Very low.			
Jain et al. (2011) [114]	VPA	BD	1250	1 month		NA. Objective: primary objective. GRADE: Very low.			
Ramakrishnappa et al. (2013) [115]	VPA	GTCS	NA	10 months		Trichogram revealed an increase in resting and dystrophic hair at the expense of growing hair. Co-occurrence between high serum levels of VPA and onset of alopecia. VPA was discontinued, and hair regrowth was observed. Objective: primary objective. GRADE: Very low.			
Panwar et al. (2016) [116]	VPA	GTCS	1000	25 days		A trichogram and punch biopsy of the scalp revealed a diagnosis of anagen effluvium. Objective: primary objective. GRADE: Very low.			
Grootens et al. (2017) [117]	VPA	BD type I	NA	1 month		The VPA-induced alopecia was observed within one month. The VPA was maintained at the same dose until the ninth month. After, biotin was started with VPA therapy. Three months later, her excessive hair loss completely disappeared. Complete hair regrowth was observed.			
Sahin et al. (2017) [47]	VPA	BD type II	1250	NA		Agomelatine (25 mg/day) was added to his therapeutical management. His hair loss stopped, and hair regrowth was observed. VPA dose was unchanged. Objective: primary objective. GRADE: Very low.			
Uygur et al. (2019) [118]	VPA	BD	1250	NA		Alopecia as a secondary effect of VPA was observed. In the ninth month, hair growth was curly. Objective: primary objective. GRADE: Very low.			
Govindan et al. (2020) [119]	VPA	BD	NA	4 months		Alopecia was observed in a breastfed infant, possibly due to the mother taking VPA. Two months after VPA discontinuation by the mother, hair regrowth in the breastfed infant was observed. A complete reversal of alopecia was observed. Objective: primary objective. GRADE: Very low.			
**Case series**									
**Reference**	**ASM**	**N of cases**	**Diagnosis**	**Dosage (mg/day)**	**Duration**	**Comment**			
Breathnach et al. (1982) [120]	CBZ	2	Trigeminal neuralgia, mood stabilizer	300	2 weeks	Transient alopecia was observed. Objective: primary objective. GRADE: Low.			
Ikeda et al. (1997) [53]	CBZ	2	Focal epilepsy	300–600	2 months	Serum CBZ levels for both patients were 5 mg/L and 6.5 mg/L when hair loss started. Objective: primary objective. GRADE: Low.			
Zou et al. (2014) [65]	LEV	5	Focal epilepsy or GTCS	500–1000	3–8 weeks	Hair loss was remitted/improved in all five patients. No relationship of LEV dose with the time of alopecia onset was observed. Objective: primary objective. GRADE: Low.			
Aghamollaii et al. (2017) [66]	LEV	3	Myoclonic epilepsy	1250–1500	2 months	In all three patients, zinc supplementation resolved alopecia despite the continuation of LEV. In one individual, the LEV dose was increased to 1750 mg/day without the occurrence of alopecia. Objective: primary objective. GRADE: Low.			
Holowach et al. (1960) [99]	TMD	2	Focal epilepsy or GTCS	900–1200	6 weeks	Hair regrowth started after TMD discontinuation. Objective: primary objective. GRADE: Low.			
Lampl et al. (1996) [96]	VGB	5	Focal epilepsy	2000	3–7 weeks	Hair regrowth was seen after 1–8 weeks of VGB discontinuation. Objective: primary objective. GRADE: Low.			
Jeavons et al. (1977) [121]	VPA	5	NA	NA	NA	Five cases of VPA-induced alopecia. Interestingly, the regrowth of hair was curly. Objective: primary objective. GRADE: Low.			
Tomita et al. (2015) [122]	VPA	3	2 BD and 1 focal epilepsy	800–1200	40 days	A patient showed hair loss improvement within 15 days of the management. The other individual improved after 84 days. Objective: primary objective. GRADE: Low.			
Thomson et al. (2017) [48]	VPA	3	GTCS	500–1500	7 months	Zinc supplementation improved hair loss in two patients within 1–2 months. Higher doses of VPA caused alopecia earlier than lower doses of VPA. Objective: primary objective. GRADE: Low.			
Cooper-Mahkorn et al. (2007) [123]	ZNS	2	Focal epilepsy	300–500	9 months	In both patients, hair loss was reversible after ZNS discontinuation. Objective: primary objective. GRADE: Low.			
**Mixed studies**									
**Reference**	**ASM**	**Study type**		**N of cases**		**Comment**			
Hirsch et al. (2018) [124]	BRV	Observational study		102		The study reported one case of alopecia. Objective: secondary objective. GRADE: Low.			
Ryvlin et al. (2022) [125]	BRV	Clinical trial		1164		The study reported one case of alopecia. Objective: secondary objective. GRADE: Low.			
Talati et al. (2011) [126]	CBZ	Systematic review		NA		No differences in the risk of alopecia were noted with newer antiseizure medications and CBZ. Newer antiseizure medications decreased the risk of experiencing alopecia when compared with VPA and when LTG and TPM were individually compared with VPA. Objective: primary objective. GRADE: High.			
Mattson et al. (1992) [52]	CBZ; VPA	Randomized controlled trial		480		Overall, failure in management was more commonly due to seizures in VPA patients and to serious side effects in CBZ individuals. Dose investigate: CBZ: 722 mg/day (mean); VPA: 2099 mg/day (mean). Objective: secondary objective. GRADE: Low.			
Richens et al. (1994) [127]	CBZ; VPA	Clinical trial		300		VPA: five cases; CBZ: one case. Objective: secondary objective. GRADE: Low.			
Pillans et al. (1995) [33]	CBZ; VPA	Systematic review		820		Reports of alopecia in the World Health Organization (WHO) database on 24 August 1994. Objective: primary objective. GRADE: High.			
Verity et al. (1995) [128]	CBZ; VPA	Clinical trial		260		VPA: five cases; CBZ: two cases. The drug therapy duration was 3 months. Objective: secondary objective. GRADE: Low.			
Steinhoff et al. (2005) [129]	CBZ; LTG; VPA	Clinical trial		239		LTG: six (4.95%) cases of alopecia; VPA: three (10%) cases of alopecia. Objective: secondary objective. GRADE: Low.			
Privitera et al. (2003) [130]	CBZ; TPM; VPA	Clinical trial		613		TPM 100 mg: four (1.9%) cases of alopecia; TPM 200 mg: two (1%) cases of alopecia; CBZ 600 mg: two (1.58%) cases of alopecia; VPA 1250 mg: 14 (17.94%) cases of alopecia. Objective: secondary objective. GRADE: Low.			
Wheless et al. (2004) [131]	CBZ; TPM; VPA	Clinical trial		613		VPA 1250 mg: two (11%) cases of alopecia. CBZ: one (4%) case of alopecia. There were no cases of TPM-induced alopecia. Objective: secondary objective. GRADE: Low.			
Donati et al. (2007) [132]	CBZ, OXC, VPA	Clinical trial		112		CBZ: one case; VPA: three cases. There was no report of OXC-induced alopecia. Objective: secondary objective. GRADE: Low.			
Koeppen et al. (1987) [133]	CLB	Clinical trial		129		The study reported one case of alopecia. The study was a double-blind, placebo-controlled crossover. Objective: secondary objective. GRADE: Low.			
Satishchandra et al. (2022) [134]	CLB	Observational study		429		The study reported one case of alopecia. Objective: secondary objective. GRADE: Low.			
Klein et al. (2022) [135]	CNB	Clinical trial		355		The study reported one case of alopecia. Objective: secondary objective. GRADE: Low.			
Sperling et al. (2020) [136]	CNB	Clinical trial		1347		The study reported fifteen cases of alopecia. Objective: secondary objective. GRADE: Low.			
Villanueva et al. (2023) [137]	CNB	Observational study		2		The study reported two cases of alopecia. Objective: secondary objective. GRADE: Low.			
Chaves et al. (2017) [138]	ESL	Cohort study		52		The study reported one case of alopecia. Objective: secondary objective. GRADE: Low.			
Galiana et al. (2017) [139]	ESL	Review		NA		Most common skin reactions were rash, alopecia, and hyperhidrosis, which occurred in 0.5–3.2% of the ESL users. Objective: secondary objective. GRADE: Low.			
Hixson et al. (2021) [140]	ESL	Clinical trial		102		ESL was taken as adjunctive therapy to LEV or LTG. Objective: secondary objective. GRADE: Low.			
Knoll et al. (1998) [141]	GBP	Observational study		12		The study reported one case of alopecia. Objective: secondary objective. GRADE: Low.			
Collins et al. (2019) [142]	GBP	Observational study		42		The study reported one case of alopecia. Objective: secondary objective. GRADE: Low.			
Runge et al. (2015) [143]	LCM	Observational analysis		571		The study reported seven cases of alopecia. Objective: secondary objective. GRADE: Low.			
Biton et al. (2003) [144]	LTG, VPA	Clinical trial		38		Three individuals developed alopecia secondary to VPA. There were no reports of LTG-induced alopecia. The drug therapy duration was 8 months. Objective: secondary objective. GRADE: Low.			
Morrell et al. (2008) [145]	LTG, VPA	Clinical trial		447		VPA: 25 (11%) cases of alopecia; LTG: three (1%) cases of alopecia. Objective: secondary objective. GRADE: Low.			
Tengstrand et al. (2010) [61]	LTG	Retrospective analysis		337		Hair loss due to LTG occurs more often in women. Specific time of alopecia onset was <1 month: 22/110; 1–6 months: 50/110; ≥6 months: 38/110. Of 337 cases, 112 individualsused LTG. Rash was the most common co-reported reaction alongside alopecia (18/337). The drug therapy duration was 1–6 months. The dose investigated was 200–500 mg/day. Objective: primary objective. GRADE: Moderate.			
Chen et al. (2015) [4]	CBZ, LEV, LTG, PGB, PHT, TPM, VPA	Retrospective analysis		1903		ASM	Number of individuals using ASM	Percentage of individuals who developed alopecia	Percentage of individuals who discontinued ASM due to alopecia
						LEV	524	0.4%	0.4%
						LTG	521	0.8%	0.6%
						PHT	404	0.3%	0.3%
						CBZ	326	0.3%	0.3%
						VPA	270	8.9%	8.2%
						TPM	230	1.7%	1.7%
						PGB	143	0.7%	0.7%
						Objective: primary objective. GRADE: Moderate.			
Herranz et al. (1988) [74]	PHB, PRM, PHT, CBZ, VPA	Observational analysis		392		PHB: one case; PRM: one case; VPA: three cases. There were no cases of alopecia associated with PHT or CBZ. Objective: secondary objective. GRADE: Low.			
Vining et al. (1987) [146]	PHB, VPA	Clinical trial		21		VPA: seven cases; PHB: one case. Objective: secondary objective. GRADE: Low.			
Harmark et al. (2011) [80]	PGB	Observational analysis		1373		Web-based intensive monitoring system based at the Netherlands Pharmacovigilance Centre Lareb. Objective: primary objective. GRADE: Low.			
Villanueva et al. (2016) [89]	PMP	Observational analysis		464		The study reported one case of alopecia. Objective: secondary objective. GRADE: Low.			
Rohracher et al. (2018) [88]	PMP	Observational analysis		2396		The study reported one case of alopecia. Objective: secondary objective. GRADE: Low.			
Kluger et al. (2009) [147]	RFM	Observational analysis		60		The study reported one case of alopecia. Objective: secondary objective. GRADE: Low.			
Tan et al. (2017) [148]	RFM	Observational analysis		76		The study reported one case of alopecia. Objective: secondary objective. GRADE: Low.			
Vossler et al. (2013) [98]	TGB	Randomized controlled trial		292		The study reported one case of alopecia. Objective: secondary objective. GRADE: Low.			
Mercke et al. (2000) [8]	TGB	Review		NA		The incidence tiagabine-induced alopecia was 1%. Objective: primary objective. GRADE: Low.			
Krymchantowski et al. (2004) [149]	TPM	Randomized controlled trial		175		Alopecia was observed in one individual (3.7%). The dose investigated was 100 mg/day. Objective: secondary objective. GRADE: Low.			
Turanli et al. (2006) [150]	VGB	Observational study		111		The study reported one case of alopecia. Objective: secondary objective. GRADE: Low.			
Christe et al. (1997) [151]	VPA, OXC	Clinical trial		249		VPA: four cases. There was no case report of alopecia secondary to OXC. Objective: secondary objective. GRADE: Low.			
Jeavons et al. (1974) [152]	VPA	Clinical trial		63		All the individuals were in use of VPA and PHT. Definite hair loss was only observed in two patients. Objective: secondary objective. GRADE: Low.			
Gram et al. (1977) [153]	VPA	Clinical trial		39		The study reported two cases of alopecia. VPA dose was maintained. Objective: secondary objective. GRADE: Low.			
Hassan et al. (1979) [154]	VPA	Clinical trial		115		The study reported one case of alopecia. He had diffuse alopecia unassociated with any evidence of a primary hair or scalp disorder. The dose investigated was 400–2400 mg/day. Objective: secondary objective. GRADE: Low.			
Coulter et al. (1980) [155]	VPA	Clinical trial		100		The study reported one case of alopecia. Objective: secondary objective. GRADE: Low.			
Egger et al. (1981) [156]	VPA	Observational study		100		VPA-induced alopecia was observed in six individuals. In three of them, the hair became curly, especially in the frontal region. Additionally, a microscopic examination did not show any abnormality. Objective: secondary objective. GRADE: Low.			
Turnbull et al. (1983) [157]	VPA	Clinical trial		54		The study reported four cases of alopecia. One patient developed alopecia with plasma VPA levels of 118 and 109 p,g/mL. Objective: secondary objective. GRADE: Low.			
Spitz et al. (1991) [158]	VPA	Clinical trial		71		The VPA-induced alopecia was transient. Objective: secondary objective. GRADE: Low.			
Beydoun et al. (1997) [34]	VPA	Clinical trial		143		High doses (27 individuals) when compared to lower doses (two individuals) of VPA, were more frequently associated with alopecia. Objective: secondary objective. GRADE: Low.			
Macritchie et al. (2001) [159]	VPA	Review		NA		The VPA group had significantly more patients suffering from alopecia (RRI 143%; RR 2.43; 95% C.I. 1.05 to 5.65) than the placebo group. Objective: secondary objective. GRADE: Low.			
Schulpis et al. (2001) [160]	VPA	Clinical trial		75		VPA impaired liver mitochondrial function, resulting in low biotinidase activity. Biotin supplementation could restore some of the side effects of the drug. Objective: secondary objective. GRADE: Low.			
Ebrahimi et al. (2005) [161]	VPA	Observational study		211		Three cases were female, and three were male. The estimated frequency of hair loss was 3.5% of the VPA user population, which is lower than previously reported frequencies (6–12%). The drug therapy duration was 3 months. Objective: primary objective. GRADE: Moderate.			
Kocer et al. (2005) [162]	VPA	Observational study		62		The authors assessed the skin findings associated with the long-term use of ASMs. Alopecia was the only skin condition related to ASM usage, and it was seen in four patients (6.5%) using VPA. Objective: primary objective. GRADE: Moderate.			
Joffe et al. (2006) [163]	VPA	Randomized controlled trial		86		Among subjects who developed oligomenorrhea while taking VPA, 22% had male-pattern alopecia. Objective: secondary objective. GRADE: Low.			
McCabe et al. (2006) [164]	VPA	Clinical trial		41		Assessment of efficacy and safety of conversion from delayed-release VPA to extended-release VPA. The drug therapy duration was 3 months. Objective: secondary objective. GRADE: Low.			
Jedrzejczak et al. (2007) [165]	VPA	Clinical trial		1984		The incidence of VPA-induced alopecia was 2.2%. Seven individuals discontinued VPA due to alopecia. Objective: secondary objective. GRADE: Low.			
Yilmaz et al. (2009) [38]	VPA	Observational study		32		Findings suggest hair loss can be attributed to serum zinc levels and serum biotinidase activity depletion within the first 3 months. After 6 months, biotinidase activity returns to normal, but serum zinc levels stay depleted. The drug therapy duration was 3–6 months. Objective: primary objective. GRADE: Moderate.			
Castro-Gago et al. (2011) [166]	VPA, CBZ	Observational study		20		Hair loss was observed in three patients treated with VPA, with normal serum levels of biotin, zinc, and biotinidase activity, and the alopecia disappeared with the oral administration of biotin (10 mg/d) within three months. Objective: secondary objective. GRADE: Low.			
Han et al. (2015) [167]	VPA	Observational study		561		A partial recovery of hair growth was observed in all the individuals. Objective: primary objective. GRADE: low.			
Kompally et al. (2015) [168]	VPA	Observational study		70		VPA-induced alopecia was transient. Objective: primary objective. GRADE: Low.			
Yamak et al. (2015) [169]	VPA	Observational study		72		VPA-induced alopecia was mild in two cases, moderate in six cases, and severe in four cases. The drug therapy duration was 1 month. Objective: secondary objective. GRADE: Low.			
Druschky et al. (2018) [170]	VPA, LTG	Observational study		47,613		VPA: 16 cases; LTG: one case. Objective: primary objective. GRADE: Moderate.			
Kakunje et al. (2018) [49]	VPA	Review		NA		Hair curling can occur in about 3.5% of individuals. Topical VPA can induce hair growth. Starting with a low dose and with a progressive dose increase, VPA might counteract alopecia. The drug therapy duration was 25–90 days. The dose investigated was >800 mg/day. Objective: primary objective. GRADE: Moderate.			
Wang et al. (2019) [35]	VPA	Systematic review		25		Patients taking a lower dose (≤750 mg/d) had a similar risk of developing alopecia to patients administered a higher dose (>750 mg/d). Patients taking VPA for longer than 6 months did not increase the risk of alopecia compared to taking VPA for less than 6 months. The dose investigated was 400–2099 mg/day. Objective: primary objective. GRADE: High.			
Pruccoli et al. (2023) [171]	VPA	Observational study		NA		One patient developed alopecia due to VPA use. Objective: secondary objective. GRADE: low.			

Abbreviations: ASM, antiseizure medication; BD, bipolar disorder; BRV, brivaracetam; CBZ, carbamazepine; CLB, clobazam; CNB, cenobamate; ESL, eslicarbazepine acetate; GBP, gabapentin; GTCS, generalized tonic-clonic seizures; LCM, lacosamide; LEV, levetiracetam; LTG, lamotrigine; N, NA, not available/not reported; OXC, oxcarbazepine; PGB, pregabalin; PHB, phenobarbital; PHT, phenytoin; PMP, perampanel; PRM, primidone; RFM, rufinamide; TGB, tiagabine; TPM, topiramate; TMD, trimethadione; VGB, vigabatrin; VPA, valproic acid/valproate; ZNS, zonisamide. ^a^ Duration was characterized by the time from drug intake to alopecia onset. ^b^ The description of the alopecia being evaluated as a primary or secondary objective in the study will be included in the comments. ^c^ The GRADE (Grading of Recommendations, Assessment, Development, and Evaluations) will be described in the comments.

**Table 4:** Summary of antiseizure medication-induced alopecia.

Antiseizure Medication	Pubmed (*n*: 180) ^i^	Literature (*n*: 1656) ^ii^	Incidence, Reference
Valproate	70	983	2.2%, Jedrzejczak et al. (2007) [165] 3.5%, Ebrahimi et al. (2005) [161] 12%, Mattson et al. (1992) [52]
Carbamazepine	31	225	0.3%, Chen et al. (2015) [4] 6%, Mattson et al. (1992) [52]
Phenytoin	20	4	0.3%, Chen et al. (2015) [4]
Phenobarbital	16	5	1.2%, Herranz et al. (1988) [74]
Lamotrigine	10	355	0.8%, Chen et al. (2015) [4]
Levetiracetam	8	14	0.4%, Chen et al. (2015) [4]
Gabapentin	6	5	2.3%, Collins et al. (2019) [142]
Topiramate	5	13	1.7%, Chen et al. (2015) [4] 3.7%, Krymchantowski et al. (2004) [149]
Oxcarbazepine	4	0	NA
Clobazam	2	2	0.23%, Satishchandra et al. (2022) [134]
Felbamate	2	0	NA
Vigabatrin	2	6	0.9%, Turanli et al. (2006) [150] 9.6%, Lampl et al. (1996) [96]
Pregabalin	1	4	0.07%, Harmark et al. (2011) [80] 0.7%, Chen et al. (2015) [4]
Primidone	1	1	1.5%, Herranz et al. (1988) [74]
Trimethadione	1	2	NA
Zonisamide	1	2	NA
Brivaracetam	0	2	0.08%, Ryvlin et al. (2022) [125] 0.9%, Hirsch et al. (2018) [124]
Cenobamate	0	18	0.28%, Klein et al. (2022) [135] 1.11%, Sperling et al. (2020) [136]
Eslicarbazepine	0	3	1.92%, Chaves et al. (2017) [138] 1.93%, Hixson et al. (2021) [140]
Lacosamide	0	7	1.22%, Runge et al. (2015) [143]
Perampanel	0	2	0.04%, Rohracher et al. (2018) [88] 0.21%, Villanueva et al. (2016) [89]
Rufinamide	0	2	1.31%, Tan et al. (2017) [148] 1.66%, Kluger et al. (2009) [147]
Tiagabine	0	1	1%, Mercke et al. (2000) [8]

Abbreviations: NA, not available/not reported. (^i^) PubMed: the number represents the number of results found in PubMed with the terms [(alopecia) AND (ASM)]. (^ii^) Literature: the number represents the number of cases reported with ASM-induced alopecia in the literature.

## Data Availability

Not applicable.

## References

[1] Getnet A., Woldeyohannes S.M., Bekana L., Mekonen T., Fekadu W., Menberu M., Yimer S., Assaye A., Belete A., Belete H. (2016). Antiepileptic Drug Nonadherence and Its Predictors among People with Epilepsy. Behav. Neurol..

[2] Buck D., Jacoby A., Baker G.A., Chadwick D.W. (1997). Factors influencing compliance with antiepileptic drug regimes. Seizure.

[3] Kanner A.M. (2009). To comply with AED therapy… what patients are not told!. Epilepsy Curr..

[4] Chen B., Choi H., Hirsch L.J., Moeller J., Javed A., Kato K., Legge A., Buchsbaum R., Detyniecki K. (2015). Cosmetic side effects of antiepileptic drugs in adults with epilepsy. Epilepsy Behav..

[5] Perucca P., Gilliam F.G. (2012). Adverse effects of antiepileptic drugs. Lancet Neurol..

[6] Kinderen R.J., Evers S.M., Rinkens R., Postulart D., Vader C.I., Majoie M.H., Aldenkamp A.P. (2014). Side-effects of antiepileptic drugs: The economic burden. Seizure.

[7] Stroud J.D. (1987). Diagnosis and management of the hair loss patient. Cutis.

[8] Mercke Y., Sheng H., Khan T., Lippmann S. (2000). Hair loss in psychopharmacology. Ann. Clin. Psychiatry.

[9] Botega A.R., Amorim C.V., Teixeira F., Mello C.D., Stelini R.F., Velho P.N., Cintra M.L. (2023). Scarring versus Non-Scarring Alopecia: An Interobserver Histopathological Reproducibility Study. Ski. Appendage Disord..

[10] Llau M.E., Viraben R., Montastruc J.L. (1995). Drug-induced alopecia: Review of the literature. Therapie.

[11] Lemieux J., Maunsell E., Provencher L. (2008). Chemotherapy-induced alopecia and effects on quality of life among women with breast cancer: A literature review. Psychooncology.

[12] Can G., Demir M., Erol O., Aydiner A. (2013). A comparison of men and women’s experiences of chemotherapy-induced alopecia. Eur. J. Oncol. Nurs..

[13] Paus R., Cotsarelis G. (1999). The biology of hair follicles. N. Engl. J. Med..

[14] Lindner G., Botchkarev V.A., Botchkareva N.V., Ling G., Veen C., Paus R. (1997). Analysis of apoptosis during hair follicle regression (catagen). Am. J. Pathol..

[15] Lousada M.B., Lachnit T., Edelkamp J., Rouillé T., Ajdic D., Uchida Y., Nardo A., Bosch T.C., Paus R. (2021). Exploring the human hair follicle microbiome. Br. J. Dermatol..

[16] Natarelli N., Gahoonia N., Sivamani R.K. (2023). Integrative and Mechanistic Approach to the Hair Growth Cycle and Hair Loss. J. Clin. Med..

[17] Popa A., Carsote M., Cretoiu D., Dumitrascu M.C., Nistor C.E., Sandru F. (2023). Study of the Thyroid Profile of Patients with Alopecia. J. Clin. Med..

[18] Courtois M., Loussouarn G., Hourseau C., Grollier J.F. (1995). Ageing and hair cycles. Br. J. Dermatol..

[19] Bemt P.M., Brodie-Meijer C.C., Krijnen R.M., Nieboer C. (1999). Drug induced alopecia. Ned. Tijdschr. Geneeskd..

[20] Murad A., Maguire J., Bergfeld W. (2021). Drug-induced alopecia areata?. Clin. Exp. Dermatol..

[21] Starace M., Orlando G., Bruni F., Alessandrini A., Piraccini B.M. (2022). Anagen effluvium and the role of trichoscopy. Clin. Exp. Dermatol..

[22] Rossi A., Caro G., Fortuna M.C., Pigliacelli F., D’Arino A., Carlesimo M. (2020). Prevention and Treatment of Chemotherapy-Induced Alopecia. Dermatol. Pract. Concept..

[23] Moattari C.R., Jafferany M. (2022). Psychological Aspects of Hair Disorders: Consideration for Dermatologists, Cosmetologists, Aesthetic, and Plastic Surgeons. Ski. Appendage Disord..

[24] Tosti A., Misciali C., Piraccini B.M., Peluso A.M., Bardazzi F. (1994). Drug-induced hair loss and hair growth. Incidence, management and avoidance. Drug Saf..

[25] Trüeb R.M. (2010). Chemotherapy-induced hair loss. Ski. Ther. Lett..

[26] Malkud S. (2015). Telogen Effluvium: A Review. J. Clin. Diagn. Res..

[27] Sinclair R. (1999). Diffuse hair loss. Int. J. Dermatol..

[28] McKinney P.A., Finkenbine R.D., DeVane C.L. (1996). Alopecia and mood stabilizer therapy. Ann. Clin. Psychiatry.

[29] Johannessen C.U. (2000). Mechanisms of action of valproate: A commentatory. Neurochem. Int..

[30] Rissardo J.P., Caprara A.L., Durante Í. (2021). Valproate-associated Movement Disorder: A Literature Review. Prague Med. Rep..

[31] Schmidt D. (1984). Adverse effects of valproate. Epilepsia.

[32] Völzke E., Doose H. (1973). Dipropylacetate (Dépakine, Ergenyl) in the treatment of epilepsy. Epilepsia.

[33] Pillans P.I., Woods D.J. (1995). Drug-associated alopecia. Int. J. Dermatol..

[34] Beydoun A., Sackellares J.C., Shu V. (1997). Safety and efficacy of divalproex sodium monotherapy in partial epilepsy: A double-blind, concentration-response design clinical trial. Depakote Monotherapy for Partial Seizures Study Group. Neurology.

[35] Wang X., Wang H., Xu D., Zhu L., Liu L. (2019). Risk of valproic acid-related alopecia: A systematic review and meta-analysis. Seizure.

[36] Praharaj S.K., Munoli R.N., Udupa S.T., Vaidyanathan S. (2022). Valproate-associated hair abnormalities: Pathophysiology and management strategies. Hum. Psychopharmacol..

[37] Rebora A. (1993). Telogen effluvium: An etiopathogenetic theory. Int. J. Dermatol..

[38] Yilmaz Y., Tasdemir H.A., Paksu M.S. (2009). The influence of valproic acid treatment on hair and serum zinc levels and serum biotinidase activity. Eur. J. Paediatr. Neurol..

[39] Hurd R.W., Rinsvelt H.A., Wilder B.J., Karas B., Maenhaut W., Reu L. (1984). Selenium, zinc, and copper changes with valproic acid: Possible relation to drug side effects. Neurology.

[40] Jacobsen N.W., Halling-Sorensen B., Birkved F.K. (2008). Inhibition of human aromatase complex (CYP19) by antiepileptic drugs. Toxicol. In Vitro.

[41] Menon B., Harinarayan C.V. (2010). The effect of anti epileptic drug therapy on serum 25-hydroxyvitamin D and parameters of calcium and bone metabolism--a longitudinal study. Seizure.

[42] Uehlinger C., Barrelet L., Touabi M., Baumann P. (1992). Alopecia and mood stabilizers: Two case reports. Eur. Arch. Psychiatry Clin. Neurosci..

[43] Henriksen O., Johannessen S.I. (1982). Clinical and pharmacokinetic observations on sodium valproate—A 5-year follow-up study in 100 children with epilepsy. Acta Neurol. Scand..

[44] Fatemi S.H., Calabrese J.R. (1995). Treatment of valproate-induced alopecia. Ann. Pharmacother..

[45] Trost L.B., Bergfeld W.F., Calogeras E. (2006). The diagnosis and treatment of iron deficiency and its potential relationship to hair loss. J. Am. Acad. Dermatol..

[46] Castro-Gago M., Gómez-Lado C., Eirís-Puñal J., Díaz-Mayo I., Castiñeiras-Ramos D.E. (2010). Serum biotinidase activity in children treated with valproic acid and carbamazepine. J. Child. Neurol..

[47] Sahin E.K., Can S.S., Caykoylu A., Atagun M.I. (2017). Agomelatine may alleviate valproate induced hair loss. J. Psych. Neurol. Sci..

[48] Thomson S.R., Mamulpet V., Adiga S. (2017). Sodium Valproate Induced Alopecia: A Case Series. J. Clin. Diagn. Res..

[49] Kakunje A., Prabhu A., Priya E.S., Karkal R., Kumar P., Gupta N., Rahyanath P.K. (2018). Valproate: It’s Effects on Hair. Int. J. Trichol..

[50] Pellock J.M. (1987). Carbamazepine side effects in children and adults. Epilepsia.

[51] Shuper A., Stahl B., Weitz R. (1985). Carbamazepine-induced hair loss. Drug Intell. Clin. Pharm..

[52] Mattson R.H., Cramer J.A., Collins J.F. (1992). A comparison of valproate with carbamazepine for the treatment of complex partial seizures and secondarily generalized tonic-clonic seizures in adults. N. Engl. J. Med..

[53] Ikeda A., Shibasaki H., Shiozaki A., Kimura J. (1997). Alopecia with carbamazepine in two patients with focal seizures. J. Neurol. Neurosurg. Psychiatry.

[54] Oh S.H., Kim D.S., Kwon Y.S., Lee J.H., Lee K.H. (2008). Concurrence of palmoplantar psoriasiform eruptions and hair loss during carbamazepine treatment. Acta Derm. Venereol..

[55] Goldenberg M.M. (2010). Overview of drugs used for epilepsy and seizures: Etiology, diagnosis, and treatment. Pharm. Ther..

[56] Rybakowski J.K. (2023). Mood Stabilizers of First and Second Generation. Brain Sci..

[57] Shirazi Z., Inaloo S. (2008). Intravenous immunoglobulin in the treatment of lamotrigine- induced toxic epidermal necrolysis. Iran. J. Allergy Asthma Immunol..

[58] Patrizi A., Savoia F., Negosanti F., Posar A., Santucci M., Neri I. (2005). Telogen effluvium caused by magnesium valproate and lamotrigine. Acta Derm. Venereol..

[59] Hillemacher T., Bleich S., Kornhuber J., Frieling H. (2006). Hair loss as a side effect of lamotrigine treatment. Am. J. Psychiatry.

[60] Solmi M., Tamiello G.I., Manuli G. (2017). Lamotrigine Induces Hair Loss in a Patient with Treatment-Resistant Major Depressive Disorder. Am. J. Ther..

[61] Tengstrand M., Star K., Puijenbroek E.P., Hill R. (2010). Alopecia in association with lamotrigine use: An analysis of individual case safety reports in a global database. Drug Saf..

[62] Stephen L.J., Kelly K., Parker P., Brodie M.J. (2011). Levetiracetam monotherapy--outcomes from an epilepsy clinic. Seizure.

[63] Hovinga C.A. (2001). Levetiracetam: A novel antiepileptic drug. Pharmacotherapy.

[64] Neyens L.G., Alpherts W.C., Aldenkamp A.P. (1995). Cognitive effects of a new pyrrolidine derivative (levetiracetam) in patients with epilepsy. Prog. Neuropsychopharmacol. Biol. Psychiatry.

[65] Zou X., Hong Z., Zhou D. (2014). Hair loss with levetiracetam in five patients with epilepsy. Seizure.

[66] Aghamollaii V., Khan Z.G., Maneshi A., Ghaeli P. (2017). Role of Zinc Supplementation in the Treatment of Levetiracetam-Induced Hair Loss: A Case Series. J. Pharm. Care..

[67] Goa K.L., Sorkin E.M. (1993). Gabapentin. A review of its pharmacological properties and clinical potential in epilepsy. Drugs.

[68] Rose M.A., Kam P.C. (2002). Gabapentin: Pharmacology and its use in pain management. Anaesthesia.

[69] Eker H.E., Cok O.Y., Aribogan A. (2009). Alopecia associated with gabapentin in the treatment of neuropathic pain. J. Pain Symptom Manag..

[70] Picard C., Jonville-Bera A.P., Billard C., Autret E. (1997). Alopecia associated with gabapentin: First case. Ann. Pharmacother..

[71] Chuang Y.C., Chang W.N., Chen I.L., Yang J.Y., Ho J.C., Kuo H.W. (2002). Topiramate-induced hair loss: Case report. Dermatol. Psychosom..

[72] Ghafoor I., Hosseini H. (2017). Hair Loss Following The Topiramate Treatment. J. Babol. Univ. Med. Sci..

[73] Lagrand T.J., Lehn A.C. (2021). Tremor Drugs in the Crosshairs. Tremor Other Hyperkinetic Mov..

[74] Herranz J.L., Armijo J.A., Arteaga R. (1988). Clinical side effects of phenobarbital, primidone, phenytoin, carbamazepine, and valproate during monotherapy in children. Epilepsia.

[75] Wallace S.J. (1996). A comparative review of the adverse effects of anticonvulsants in children with epilepsy. Drug Saf..

[76] Kuhne A.C., Pitta A.C., Galassi S.C., Gonçalves A.M., Cardoso A.C., Paz J.A., Campos L.M., Silva C.A. (2019). Munchausen by proxy syndrome mimicking childhood-onset systemic lupus erythematosus. Lupus.

[77] Mangalvedhekar S.S., Gogtay N.J., Manjula S., Kadam V.S., Dalvi S.S., Shah P.U., Badakere S.S., Pradhan V.D., Kshirsagar N.A. (2001). Phenytoin associated alopecia: Drug induced lupus. J. Assoc. Physicians India.

[78] Neki N.S., Shah D.M. (2015). Phenytoin induced alopecia & Lupus: A case report. RGUHS J. Med. Sci..

[79] Onaolapo A.Y., Adebayo A.A., Onaolapo O.J. (2018). Oral phenytoin protects against experimental cyclophosphamide-chemotherapy induced hair loss. Pathophysiology.

[80] Harmark L., Puijenbroek E., Straus S., Grootheest K. (2011). Intensive monitoring of pregabalin: Results from an observational, Web-based, prospective cohort study in the Netherlands using patients as a source of information. Drug Saf..

[81] Morse D.C., Henck J.W., Bailey S.A. (2016). Developmental Toxicity Studies with Pregabalin in Rats: Significance of Alterations in Skull Bone Morphology. Birth Defects Res. B Dev. Reprod. Toxicol..

[82] Turgut C., İzki A.A. (2020). Hair loss due to pregabaline: A case report. Med. Res. Rep..

[83] Suh J.H., Oh W.J., Park K.Y., Seo S.J., Hong C.K. (2016). DRESS syndrome induced by pregabalin in postherpetic neuralgia. Korean J. Derm..

[84] Franco V., Crema F., Iudice A., Zaccara G., Grillo E. (2013). Novel treatment options for epilepsy: Focus on perampanel. Pharmacol. Res..

[85] French J.A., Krauss G.L., Biton V., Squillacote D., Yang H., Laurenza A., Kumar D., Rogawski M.A. (2012). Adjunctive perampanel for refractory partial-onset seizures: Randomized phase III study 304. Neurology.

[86] French J.A., Krauss G.L., Wechsler R.T., Wang X.F., DiVentura B., Brandt C., Trinka E., O’Brien T.J., Laurenza A., Patten A. (2015). Perampanel for tonic-clonic seizures in idiopathic generalized epilepsy A randomized trial. Neurology.

[87] Lin K.L., Lin J.J., Chou M.L., Hung P.C., Hsieh M.Y., Chou I.J., Lim S.N., Wu T., Wang H.S. (2018). Efficacy and tolerability of perampanel in children and adolescents with pharmacoresistant epilepsy: The first real-world evaluation in Asian pediatric neurology clinics. Epilepsy Behav..

[88] Rohracher A., Zimmermann G., Villanueva V., Garamendi I., Sander J.W., Wehner T., Shankar R., Ben-Menachem E., Brodie M.J., Pensel M.C. (2018). Perampanel in routine clinical use across Europe: Pooled, multicenter, observational data. Epilepsia.

[89] Villanueva V., Garcés M., López-González F.J., Rodriguez-Osorio X., Toledo M., Salas-Puig J., González-Cuevas M., Campos D., Serratosa J.M., González-Giráldez B. (2016). Safety, efficacy and outcome-related factors of perampanel over 12 months in a real-world setting: The FYDATA study. Epilepsy Res..

[90] Johansen T.N., Greenwood J.R., Frydenvang K., Madsen U., Krogsgaard-Larsen P. (2003). Stereostructure-activity studies on agonists at the AMPA and kainate subtypes of ionotropic glutamate receptors. Chirality.

[91] Jara C.P., Berti B.A., Mendes N.F., Engel D.F., Zanesco A.M., Souza G.F., Bezerra R.M., Bagatin J.T., Maria-Engler S.S., Morari J. (2021). Glutamic acid promotes hair growth in mice. Sci. Rep..

[92] Huang Y.L., Hsieh M.Y., Hsiao P.F., Sheen J.M., Yu H.R., Kuo H.C., Chen S.T., Huang J.L., Yang K.D., Lee W.I. (2009). Alopecia areata universalis after phenobarbital-induced anti-convulsant hypersensitivity syndrome. Immunol. Investig..

[93] Knutsen A.P., Shah M., Schwarz K.B., Tsai C.C. (1986). Graft versus host-like illness in a child with phenobarbital hypersensitivity. Pediatrics.

[94] Bavdekar S.B., Muranjan M.N., Gogtay N.J., Kantharia V., Kshirsagar N.A. (2004). Anticonvulsant hypersensitivity syndrome: Lymphocyte toxicity assay for the confirmation of diagnosis and risk assessment. Ann. Pharmacother..

[95] Ghorani-Azam A., Balali-Mood M., Riahi-Zanjani B., Darchini-Maragheh E., Sadeghi M. (2021). Acute Phenobarbital Poisoning for the Management of Seizures in Newborns and Children; A Systematic Literature Review. CNS Neurol. Disord. Drug Targets.

[96] Lampl Y., Gilad R., Sarova-Pinchas I., Barak Y. (1996). Hair loss-an adverse reaction to treatment with vigabatrin. Acta Therap..

[97] Graham D. (1989). Neuropathology of vigabatrin. Br. J. Clin. Pharmacol..

[98] Vossler D.G., Morris G.L., Harden C.L., Montouris G., Faught E., Kanner A.M., Fix A., French J.A. (2013). Tiagabine in clinical practice: Effects on seizure control and behavior. Epilepsy Behav..

[99] Holowach J., Sanden H.V. (1960). Alopecia as a side effect of treatment of epilepsy with trimethadione: Report of two cases. N. Engl. J. Med..

[100] Wadhwa S. (1997). Carbamazepine Induced Hypersensitivity Syndrome with Alopecia. J. Assoc. Physic. Ind..

[101] Kohno Y., Ishii A., Shoji S. (2004). A case of hair loss induced by carbamazepine. Rinsho Shinkeigaku.

[102] Zenkov L.R. (2008). Alopecia associated with treatment of symptomatic focal epilepsy with carbamazepine. Neurol. J..

[103] Kenyon K., Mintzer S., Nei M. (2014). Carbamazepine treatment of generalized tonic-clonic seizures in idiopathic generalized epilepsy. Seizure.

[104] Rathore C., Rawat K.S., Prakash S., Rana K. (2021). Carbamazepine-Induced Acute Alopecia Areata. Neurology.

[105] Chen C.M., Chen J.Y., Chen K.T., Chu C.C., Tzeng J.I., Lan K.M. (2010). A Case Report-Alopecia as a Rare but Possible Side Effect of Gabapentin. Chin. J. Pain.

[106] Calabro R.S., Bramanti P., Spina E., Italiano D. (2013). Can zinc depletion play a role in LEV-induced hair loss? Considerations from a case study. Epilepsy Behav..

[107] Hamd R.S., Hasbini D.A. (2018). Adolescent’s Hair Loss due to Levetiracetam. J. Pediatr. Epilepsy.

[108] Missori P., Currà A. (2023). Reversible subacute hair loss induced by levetiracetam. Neurol. Sci..

[109] Krivda L.K., Campagna L.J., Mignano M.S., Cho C.S. (2022). Prolonged Drug-Induced Hypersensitivity Syndrome/DRESS with Alopecia Areata and Autoimmune Thyroiditis. Fed. Pract..

[110] Laljee H.C., Parsonage M.J. (1980). Unwanted effects of sodium valproate (Epilim) in the treatment of adult patients with epilepsy. R. Soc. Med.

[111] Khan T.A., Sheng H., Mercke Y.K., Lippmann S.B. (1999). Divalproex-induced alopecia: A case report. Psychiatr. Serv..

[112] Cinbis M., Parlaz N. (2007). A case of alopecia areata associated with low dosage VPA treatment. Eur. J. Paediatr. Neurol..

[113] Wilting I., Laarhoven J.H., Koning-Verest I.F., Egberts A.C. (2007). Valproic acid-induced hair-texture changes in a white woman. Epilepsia.

[114] Jain S., Beste B. (2011). Valproate-induced hair loss: What to tell patients. Curr. Psychiatry.

[115] Ramakrishnappa S.K., Belhekar M.N. (2013). Serum drug level-related sodium valproate-induced hair loss. Indian J. Pharmacol..

[116] Panwar J.B., Ishwar C., Bhardwaj B.L., Tilakraj R., Bansal R. (2016). Sodium valproate induced alopecia in a patient of epilepsy. J. Dent. Med. Sci..

[117] Grootens K.P., Hartong E.G. (2017). A Case Report of Biotin Treatment for Valproate-Induced Hair Loss. J. Clin. Psychiatry.

[118] Uygur Ö.F., Uygur H. (2019). Valproate Induced Hair Loss and Curly Hair in Bipolar Disorder. Clin. Psychopharmacol. Neurosci..

[119] Govindan K., Mandadi G.D. (2021). Alopecia in Breastfed Infant Possibly Due to Mother Getting Valproate. Indian J. Pediatr..

[120] Breathnach S.M., McGibbon D.H., Ive F.A., Black M.M. (1982). Carbamazepine (‘Tegretol’) and toxic epidermal necrolysis: Report of three cases with histopathological observations. Clin. Exp. Dermatol..

[121] Jeavons P.M., Clark J.E., Harding G.F. (1977). Valproate and curly hair. Lancet.

[122] Tomita T., Goto H., Yoshida T., Tanaka K., Sumiya K., Kohda Y. (2015). Dose-dependent valproate-induced alopecia in patients with mental disorders. Indian J. Pharmacol..

[123] Cooper-Mahkorn D., Bauer J. (2007). Alopecia areata during treatment with zonisamide—Two case reports. Aktuelle Neurol..

[124] Hirsch M., Hintz M., Specht A., Schulze-Bonhage A. (2018). Tolerability, efficacy and retention rate of Brivaracetam in patients previously treated with Levetiracetam: A monocenter retrospective outcome analysis. Seizure.

[125] Ryvlin P., Dimova S., Elmoufti S., Floricel F., Laloyaux C., Nondonfaz X., Biton V. (2022). Tolerability and efficacy of adjunctive brivaracetam in adults with focal seizures by concomitant antiseizure medication use: Pooled results from three phase 3 trials. Epilepsia.

[126] Talati R., Scholle J.M., Phung O.J. (2011). Effectiveness and Safety of Antiepileptic Medications in Patients with Epilepsy [Internet].

[127] Richens A., Davidson D.L., Cartlidge N.E., Easter D.J. (1994). A multicentre comparative trial of sodium valproate and carbamazepine in adult onset epilepsy. J. Neurol. Neurosurg. Psychiatry.

[128] Verity C.M., Hosking G., Easter D.J. (1995). A multicentre comparative trial of sodium valproate and carbamazepine in paediatric epilepsy. Dev. Med. Child. Neurol..

[129] Steinhoff B.J., Ueberall M.A., Siemes H., Kurlemann G., Schmitz B., Bergmann L. (2005). The LAM-SAFE Study: Lamotrigine versus carbamazepine or valproic acid in newly diagnosed focal and generalised epilepsies in adolescents and adults. Seizure.

[130] Privitera M.D., Brodie M.J., Mattson R.H., Chadwick D.W., Neto W., Wang S. (2003). Topiramate, carbamazepine and valproate monotherapy: Double-blind comparison in newly diagnosed epilepsy. Acta Neurol. Scand..

[131] Wheless J.W., Neto W., Wang S. (2004). Topiramate, carbamazepine, and valproate monotherapy: Double-blind comparison in children with newly diagnosed epilepsy. J. Child Neurol..

[132] Donati F., Gobbi G., Campistol J., Rapatz G., Daehler M., Sturm Y., Aldenkamp A.P. (2007). The cognitive effects of oxcarbazepine versus carbamazepine or valproate in newly diagnosed children with partial seizures. Seizure.

[133] Koeppen D., Baruzzi A., Capozza M., Chauvel P., Courjon J., Favel P., Harmant J., Lorenz H., Oller F.V., Procaccianti G. (1987). Clobazam in therapy-resistant patients with partial epilepsy: A double-blind placebo-controlled crossover study. Epilepsia.

[134] Satishchandra P., Rathore C., Apte A., Kumar A., Mandal A., Chauhan D., Agadi J., Gurumukhani J., Asokan K., Venkateshwarlu K. (2022). Evaluation of one-year effectiveness of clobazam as an add-on therapy to anticonvulsant monotherapy in participants with epilepsy having uncontrolled seizure episodes: An Indian experience. Epilepsy Behav..

[135] Klein P., Aboumatar S., Brandt C., Dong F., Krauss G.L., Mizne S., Sanchez-Alvarez J.C., Steinhoff B.J., Villanueva V. (2022). Long-Term Efficacy and Safety From an Open-Label Extension of Adjunctive Cenobamate in Patients with Uncontrolled Focal Seizures. Neurology.

[136] Sperling M.R., Klein P., Aboumatar S., Gelfand M., Halford J.J., Krauss G.L., Rosenfeld W.E., Vossler D.G., Wechsler R., Borchert L. (2020). Cenobamate (YKP3089) as adjunctive treatment for uncontrolled focal seizures in a large, phase 3, multicenter, open-label safety study. Epilepsia.

[137] Villanueva V., Santos-Carrasco D., Cabezudo-García P., Gómez-Ibáñez A., Garcés M., Serrano-Castro P., Castro-Vilanova M.D., Sayas D., Lopez-Gonzalez F.J., Rodríguez-Osorio X. (2023). Real-world safety and effectiveness of cenobamate in patients with focal onset seizures: Outcomes from an Expanded Access Program. Epilepsia Open.

[138] Chaves J., Breia P., Pimentel J., Pelejão R., Carvalho M., Mateus P., Grebe H., Mestre A., Fernandes H., Sousa R. (2017). Eslicarbazepine acetate as adjunctive therapy in clinical practice: ESLADOBA study. Acta Neurol. Scand..

[139] Galiana G.L., Gauthier A.C., Mattson R.H. (2017). Eslicarbazepine Acetate: A New Improvement on a Classic Drug Family for the Treatment of Partial-Onset Seizures. Drugs R D.

[140] Hixson J., Gidal B., Pikalov A., Zhang Y., Mehta D., Blum D., Cantu D., Grinnell T. (2021). Efficacy and safety of eslicarbazepine acetate as a first or later adjunctive therapy in patients with focal seizures. Epilepsy Res..

[141] Knoll J., Stegman K., Suppes T. (1998). Clinical experience using gabapentin adjunctively in patients with a history of mania or hypomania. J. Affect. Disord..

[142] Collins A., Mannion R., Broderick A., Hussey S., Devins M., Bourke B. (2019). Gabapentin for the treatment of pain manifestations in children with severe neurological impairment: A single-centre retrospective review. BMJ Paediatr. Open.

[143] Runge U., Arnold S., Brandt C., Reinhardt F., Kühn F., Isensee K., Ramirez F., Dedeken P., Lauterbach T., Noack-Rink M. (2015). A noninterventional study evaluating the effectiveness and safety of lacosamide added to monotherapy in patients with epilepsy with partial-onset seizures in daily clinical practice: The VITOBA study. Epilepsia.

[144] Biton V., Levisohn P., Hoyler S., Vuong A., Hammer A.E. (2003). Lamotrigine versus valproate monotherapy-associated weight change in adolescents with epilepsy: Results from a post hoc analysis of a randomized, double-blind clinical trial. J. Child Neurol..

[145] Morrell M.J., Hayes F.J., Sluss P.M., Adams J.M., Bhatt M., Ozkara C., Warnock C.R., Isojärvi J. (2008). Hyperandrogenism, ovulatory dysfunction, and polycystic ovary syndrome with valproate versus lamotrigine. Ann. Neurol..

[146] Vining E.P., Mellitis E.D., Dorsen M.M., Cataldo M.F., Quaskey S.A., Spielberg S.P., Freeman J.M. (1987). Psychologic and behavioral effects of antiepileptic drugs in children: A double-blind comparison between phenobarbital and valproic acid. Pediatrics.

[147] Kluger G., Kurlemann G., Haberlandt E., Ernst J.P., Runge U., Schneider F., Makowski C., Boor R., Bast T. (2009). Effectiveness and tolerability of rufinamide in children and adults with refractory epilepsy: First European experience. Epilepsy Behav..

[148] Tan H.J., Awadh M., O’Regan M., Martland T.R., Kneen R. (2017). Effectiveness and Tolerability of Rufinamide in Children and Young People: A Survey of Experience from the United Kingdom. J. Pediatr. Epilepsy.

[149] Krymchantowski A., Tavares C. (2004). Weight variations in patients receiving topiramate migraine prophylaxis in a tertiary care setting. MedGenMed.

[150] Turanli G., Celebi A., Yalnizoğlu D., Topçu M., Topaloğlu H., Banu A., Aysun S. (2006). Vigabatrin in pediatric patients with refractory epilepsy. Turk. J. Pediatr..

[151] Christe W., Krämer G., Vigonius U., Pohlmann H., Steinhoff B.J., Brodie M.J., Moore A. (1997). A double-blind controlled clinical trial: Oxcarbazepine versus sodium valproate in adults with newly diagnosed epilepsy. Epilepsy Res..

[152] Jeavons P.M., Clark J.E. (1974). Sodium valproate in treatment of epilepsy. Br. Med. J..

[153] Gram L., Wulff K., Rasmussen K.E., Flachs H., Würtz-Jorgensen A., Sommerbeck K.W., Lohren V. (1977). Valproate sodium: A controlled clinical trial including monitoring of drug levels. Epilepsia.

[154] Hassan M.N., Laljee H.C., Parsonage M.J. (1976). Sodium valproate in the treatment of resistant epilepsy. Acta Neurol. Scand..

[155] Coulter D.L., Wu H., Allen R.J. (1980). Valproic acid therapy in childhood epilepsy. JAMA.

[156] Egger J., Brett E.M. (1981). Effects of sodium valproate in 100 children with special reference to weight. Br. Med. J..

[157] Turnbull D.M., Rawlins M.D., Weightman D., Chadwick D.W. (1983). Plasma concentrations of sodium valproate: Their clinical value. Ann. Neurol..

[158] Spitz M.C., Deasy D.N. (1991). Conversion to valproate monotheraphy in nonretarded adults with primary Generalized tonic-clonic seizures. J. Epilepsy.

[159] Macritchie K.A., Geddes J.R., Scott J., Haslam D.R., Goodwin G.M. (2001). Valproic acid, valproate and divalproex in the maintenance treatment of bipolar disorder. Cochrane Database Syst. Rev..

[160] Schulpis K.H., Karikas G.A., Tjamouranis J., Regoutas S., Tsakiris S. (2001). Low serum biotinidase activity in children with valproic acid monotherapy. Epilepsia.

[161] Ebrahimi H., Shamsadini S., Eshkavari S.E. (2005). Frequency of Sodium Valproate-Induced Hair Loss and Curly Hair. Iran. J. Pharmacol. Ther..

[162] Kocer A., Sasmaz S., Ince N., Kutlar M., Cagirici S. (2005). Skin findings related to chronic usage of anti-epileptic drugs. Neurosci. (Riyadh).

[163] Joffe H., Cohen L.S., Suppes T., McLaughlin W.L., Lavori P., Adams J.M., Hwang C.H., Hall J.E., Sachs G.S. (2006). Valproate is associated with new-onset oligoamenorrhea with hyperandrogenism in women with bipolar disorder. Biol. Psychiatry.

[164] McCabe P.H., Michel N.C., McNew C.D., Lehman E.B. (2006). Conversion from delayed-release sodium valproate to extended-release sodium valproate: Initial results and long-term follow-up. Epilepsy Behav..

[165] Jedrzejczak J., Kuncíková M., Magureanu S. (2008). An observational study of first-line valproate monotherapy in focal epilepsy. Eur. J. Neurol..

[166] Castro-Gago M., Pérez-Gay L., Gómez-Lado C., Castiñeiras-Ramos D.E., Otero-Martínez S., Rodríguez-Segade S. (2011). The influence of valproic acid and carbamazepine treatment on serum biotin and zinc levels and on biotinidase activity. J. Child Neurol..

[167] Han X.N., Ma L., Zhang H.L., You H.S. (2015). Analysis of 59 cases of adverse drug reactions induced by valproate sodium. Chin. Hosp. Pharm. J..

[168] Kompally D.V., Ananthula K., Adla N., Rajesh V. (2015). Prospective Observational Study of Sodium Valproate in Seizure Control and Associated Adverse Drug Reactions in Pediatric Population. J. Dent. Med. Sci..

[169] Yamak W.R., Hmaimess G., Makke Y., Sabbagh S., Arabi M., Beydoun A., Nasreddine W. (2015). Valproate-induced enuresis: A prospective study. Dev. Med. Child Neurol..

[170] Druschky K., Bleich S., Grohmann R., Burda K., Frieling H., Hillemacher T., Neyazi A., Stübner S., Toto S. (2018). Severe hair loss associated with psychotropic drugs in psychiatric inpatients-Data from an observational pharmacovigilance program in German-speaking countries. Eur. Psychiatry.

[171] Pruccoli J., Parmeggiani A. (2023). The Role of Mood Stabilizers in Children and Adolescents with Anorexia Nervosa: A 1-year Follow-Up, Propensity Score-Matched Study. Pharmacopsychiatry.

[172] Liparoti G., Burchiani B., Mencaroni E., Tripodi D., Cara G., Verrotti A. (2022). Individualizing doses of antiepileptic drugs. Expert Opin. Drug Metab. Toxicol..

[173] Asghar F., Shamim N., Farooque U., Sheikh H., Aqeel R. (2020). Telogen Effluvium: A Review of the Literature. Cureus.

[174] Yin G.O., Siong-See J.L., Wang E.C. (2021). Telogen Effluvium—A review of the science and current obstacles. J. Dermatol. Sci..

[175] Park S.H., Seol J.E., Kim D.H., Kim H. (2020). Analysis of Microscopic Examination of Pulled Out Hair in Telogen Effluvium Patients. Ann. Dermatol..

[176] McDonald K.A., Shelley A.J., Colantonio S., Beecker J. (2017). Hair pull test: Evidence-based update and revision of guidelines. J. Am. Acad. Dermatol..

[177] Poonia K., Thami G.P., Bhalla M., Jaiswal S., Sandhu J. (2019). NonScarring Diffuse Hair Loss in Women: A Clinico-Etiological Study from tertiary care center in North-West India. J. Cosmet. Dermatol..

[178] Grover C., Khurana A. (2013). Telogen effluvium. Indian J. Dermatol. Venereol. Leprol..

[179] Harrison S., Sinclair R. (2002). Telogen effluvium. Clin. Exp. Dermatol..

[180] Kanwar A.J., Narang T. (2013). Anagen effluvium. Indian J. Dermatol. Venereol. Leprol..

[181] Liyanage D., Sinclair R. (2016). Telogen Effluvium. Cosmetics.

[182] Dhurat R., Saraogi P. (2009). Hair evaluation methods: Merits and demerits. Int. J. Trichol..

[183] Springer K., Brown M., Stulberg D.L. (2003). Common hair loss disorders. Am. Fam. Physician.

[184] Hoffmann A., Waśkiel-Burnat A., Żółkiewicz J., Blicharz L., Rakowska A., Goldust M., Olszewska M., Rudnicka L. (2021). Pili Torti: A Feature of Numerous Congenital and Acquired Conditions. J. Clin. Med..

[185] Almohanna H.M., Ahmed A.A., Tsatalis J.P., Tosti A. (2019). The Role of Vitamins and Minerals in Hair Loss: A Review. Dermatol. Ther..

[186] Slonim A.E., Sadick N., Pugliese M., Meyers-Seifer C.H. (1992). Clinical response of alopecia, trichorrhexis nodosa, and dry, scaly skin to zinc supplementation. J. Pediatr..

[187] Suzuki T., Koizumi J., Moroji T., Shiraishi H., Hori T., Baba A., Kawai N., Tada K. (1992). Effects of long-term anticonvulsant therapy on copper, zinc, and magnesium in hair and serum of epileptics. Biol. Psychiatry.

[188] Kuzuya T., Hasegawa T., Shimizu K., Nabeshima T. (1993). Effect of anti-epileptic drugs on serum zinc and copper concentrations in epileptic patients. Int. J. Clin. Pharmacol. Ther. Toxicol..

[189] Asadi-Pooya A.A., Rostaminejad M., Zeraatpisheh Z., Damabi N.M. (2021). Cosmetic adverse effects of antiseizure medications; A systematic review. Seizure.

